# Supplementary material for: Quantitative and multiplexed chemical-genetic phenotyping in mammalian cells with QMAP-Seq
Source: Nat Commun. 2020 Nov 12;11:5722. doi: 10.1038/s41467-020-19553-8 (PMC7661543; doi:10.1038/s41467-020-19553-8)
Supplement: Supplementary file 1 — Supplementary Information [file 41467_2020_19553_MOESM1_ESM.pdf]

Quantitative and multiplexed chemical-genetic phenotyping in mammalian cells with QMAP-Seq

Sonia Brockway, Geng Wang, Jasen M. Jackson, David R. Amici, Seesha R. Takagishi, Matthew R. Clutter, Elizabeth T. Bartom, Marc L. Mendillo

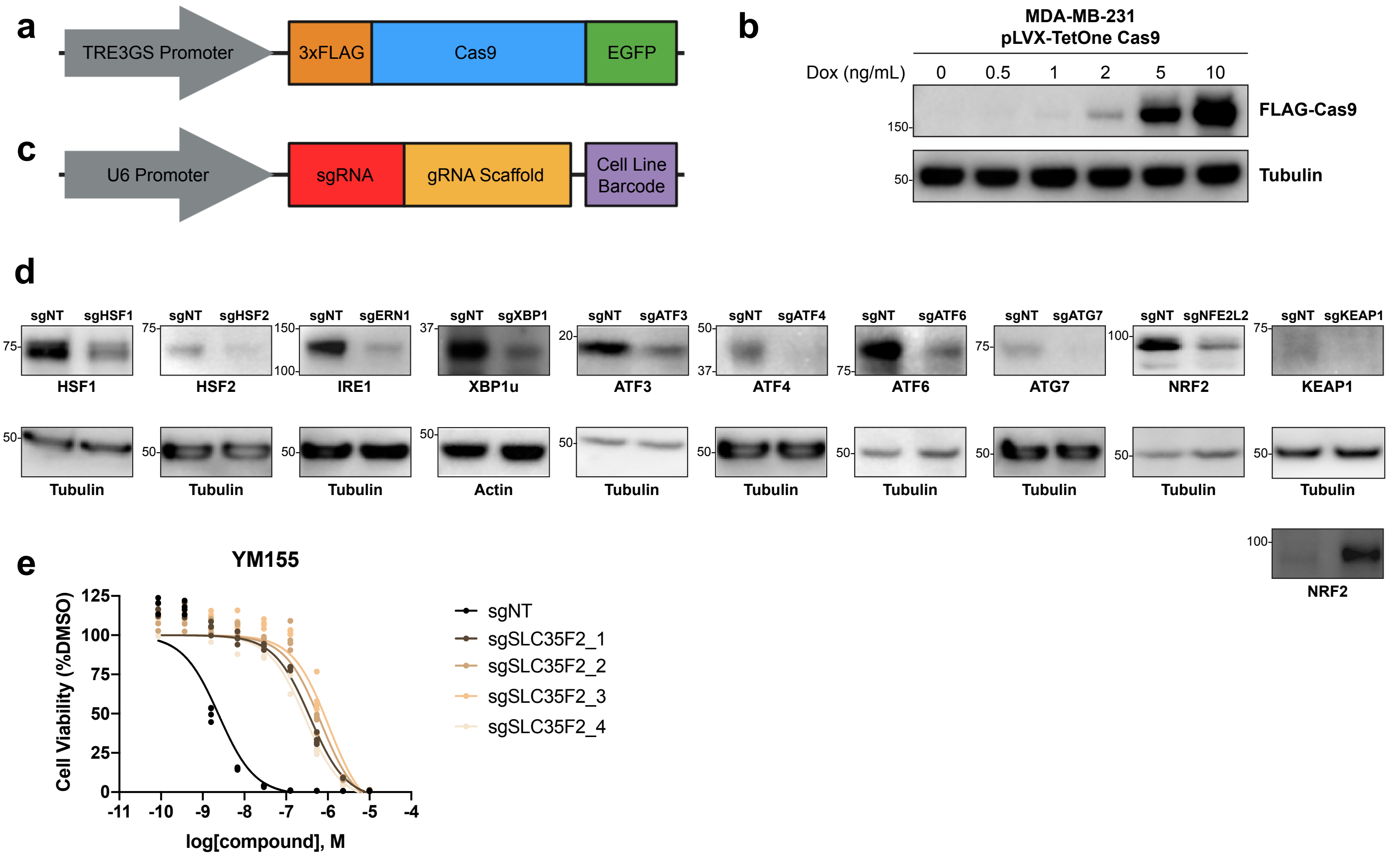

Supplementary Fig. 1. Engineering barcoded breast cancer cell lines with inducible single gene knockouts. a Schematic of doxycycline-inducible Cas9 plasmid (pLVX-TetOne Cas9). **b** MDA-MB-231 cells transduced with pLVX-TetOne Cas9 were treated with doxycycline for 48 hours. Cells were then harvested for Western blot analysis with FLAG antibody and Tubulin antibody (loading control). Results are representative of two independent experiments. Uncropped blots are provided as a Source Data file. **c** Schematic of lentiGuide-Puro plasmid engineered with an 8 bp cell line barcode downstream of the gRNA scaffold. **d** MDA-MB-231 cells that stably express pLVX-TetOne Cas9 were transduced with the indicated sgRNAs expressed from lentiGuide-Puro. Cells were treated with 10 ng/mL doxycycline for 96 hours to induce Cas9 expression and then harvested for Western blot analysis with the indicated antibodies. Experiment was performed once. Uncropped blots are provided as a Source Data file. **e** Dose-response curves as measured using CellTiter-Glo for MDA-MB-231 sgNT and sgSLC35F2 cells 72 hours after treatment with YM155. Each data point represents one of four biologically independent replicates.

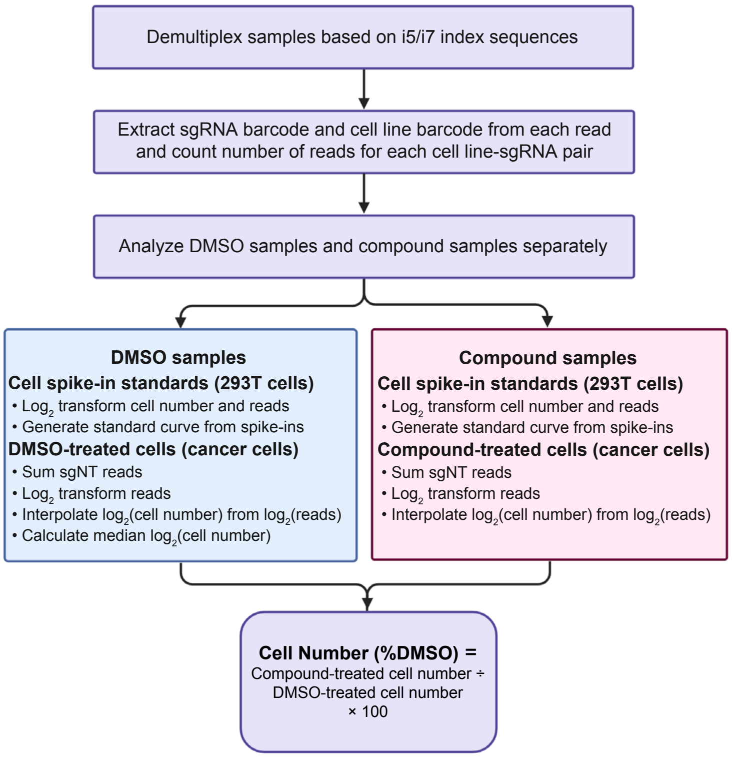

Supplementary Fig. 2. QMAP-Seq bioinformatic analysis pipeline. Pipeline for demultiplexing samples, counting sequencing reads for cell line-sgRNA pairs, interpolating cell number from sequencing reads, and calculating cell number in the presence of compound relative to DMSO.

	Positive Controls	Chemotherapy	Targeted (NCI)	Targeted (OncoKB)	Diverse (Informer Set)	Proteostasis Compounds
**Source**	N/A	cancer.org/cancer/breast-cancer/treatment/chemotherapy-for-breast-cancer.html	cancer.gov/about-cancer/treatment/types/targeted-therapies/targeted-therapies-fact-sheet	oncokb.org/dataAccess	cancerdiscovery.aacrjournals.org/content/5/11/1210.long	N/A
**Starting Number of Compounds**	4	15	16	188	481	26
**Filtering Criteria**	N/A	Part of FDA or clinical library. One compound per class.	Part of FDA or clinical library.	Part of FDA or clinical library. OncoKB levels 1-4 for indications of breast cancer, all solid tumors, or all tumors. Genetic alteration exists in breast cancer.	Part of FDA or clinical library.	Part of FDA or clinical library. Up to two compounds per target.
**Final Number of Compounds**	**4**	**9**	**8**	**13**	**154**	**12**

Supplementary Fig. 3. Selecting compounds for QMAP-Seq. Filtering criteria for selecting compounds for QMAP-Seq with multiple cell lines. Redundancies between the categories were removed to arrive at the final collection of 180 compounds.

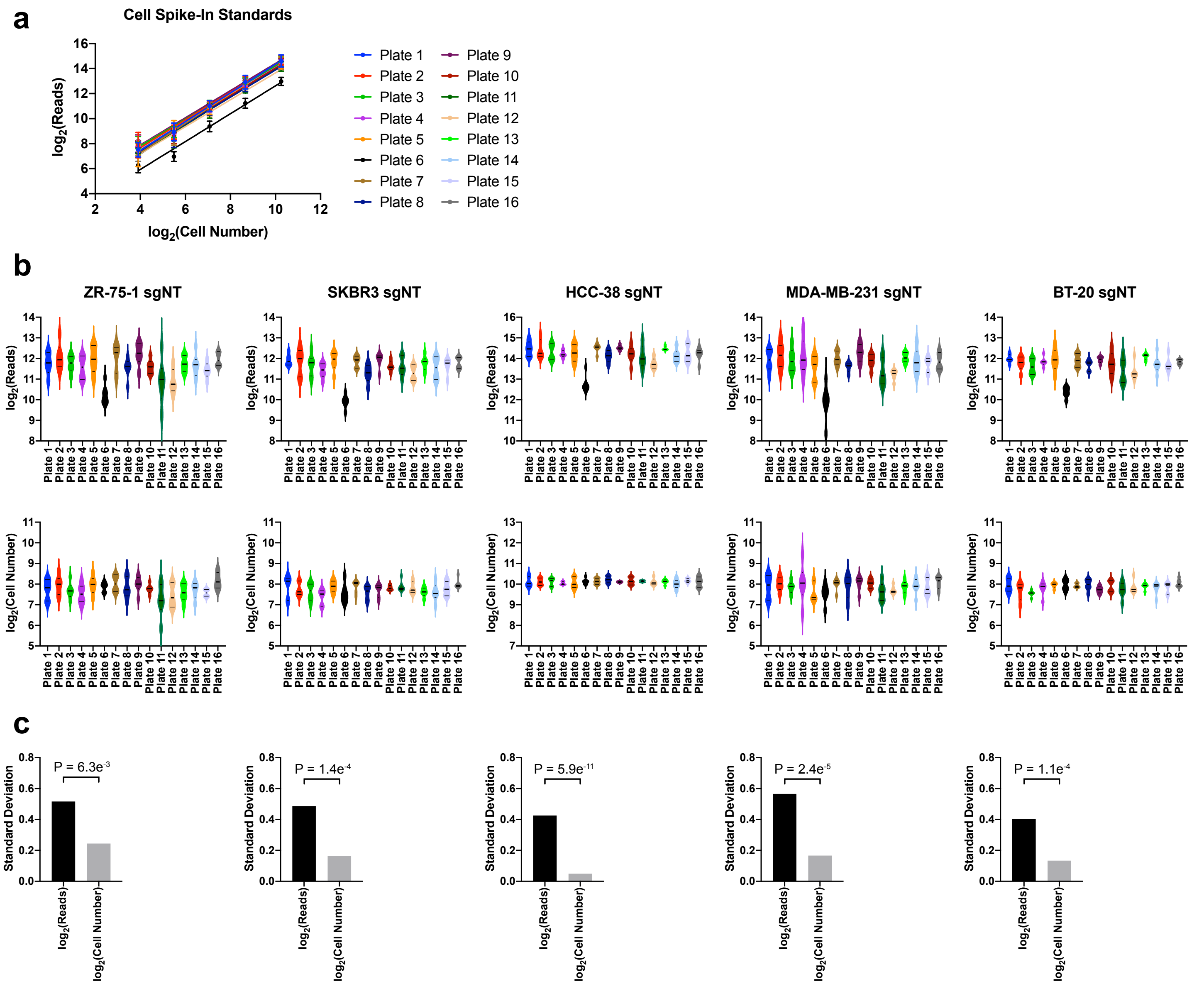

Supplementary Fig. 4. Cell spike-in standards reduce technical variation between plates. a Standard curves generated from five uniquely barcoded 293T cell spike-in standards introduced at known cell numbers. Data are represented as the mean number of sequencing reads across the six DMSO samples on a plate ± standard deviation. **b** Top: Violin plots indicating the distribution of sgNT sequencing reads for each cell line across the six DMSO samples on a plate. Bottom: Violin plots indicating the distribution of sgNT interpolated cell number for each cell line across the six DMSO samples on a plate. Thick black line denotes median. Thin black lines denote quartiles. **c** Standard deviation of sgNT sequencing reads between plates versus standard deviation of sgNT interpolated cell number between plates. Statistical significance was determined using an unpaired, two-tailed F test to compare variances (n = 16 plates).

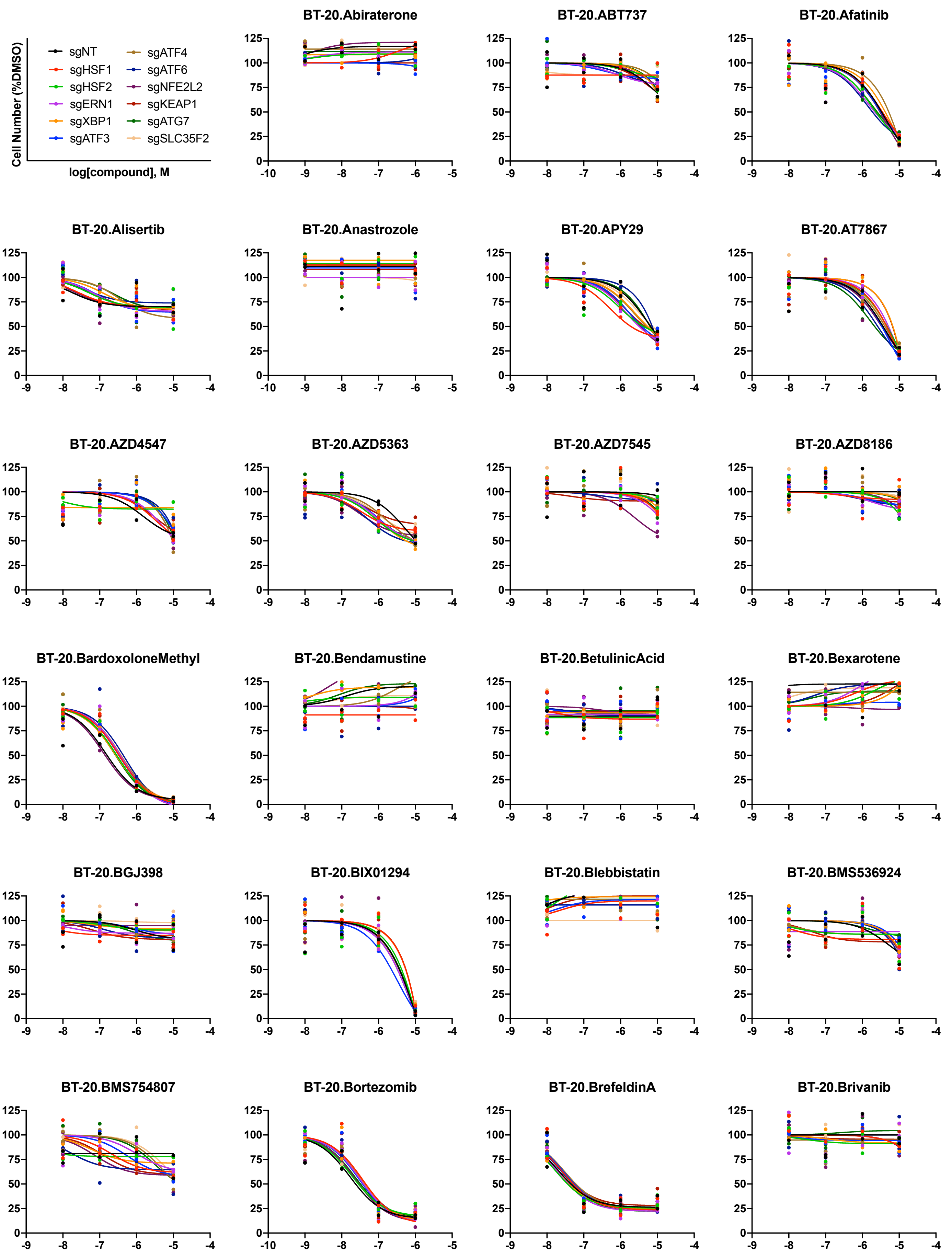

Supplementary Fig. 5. Dose-response curves generated using QMAP-Seq. Each plot represents a unique cell line-compound combination. Each data point represents one of two biologically independent replicates for the indicated sgRNA.

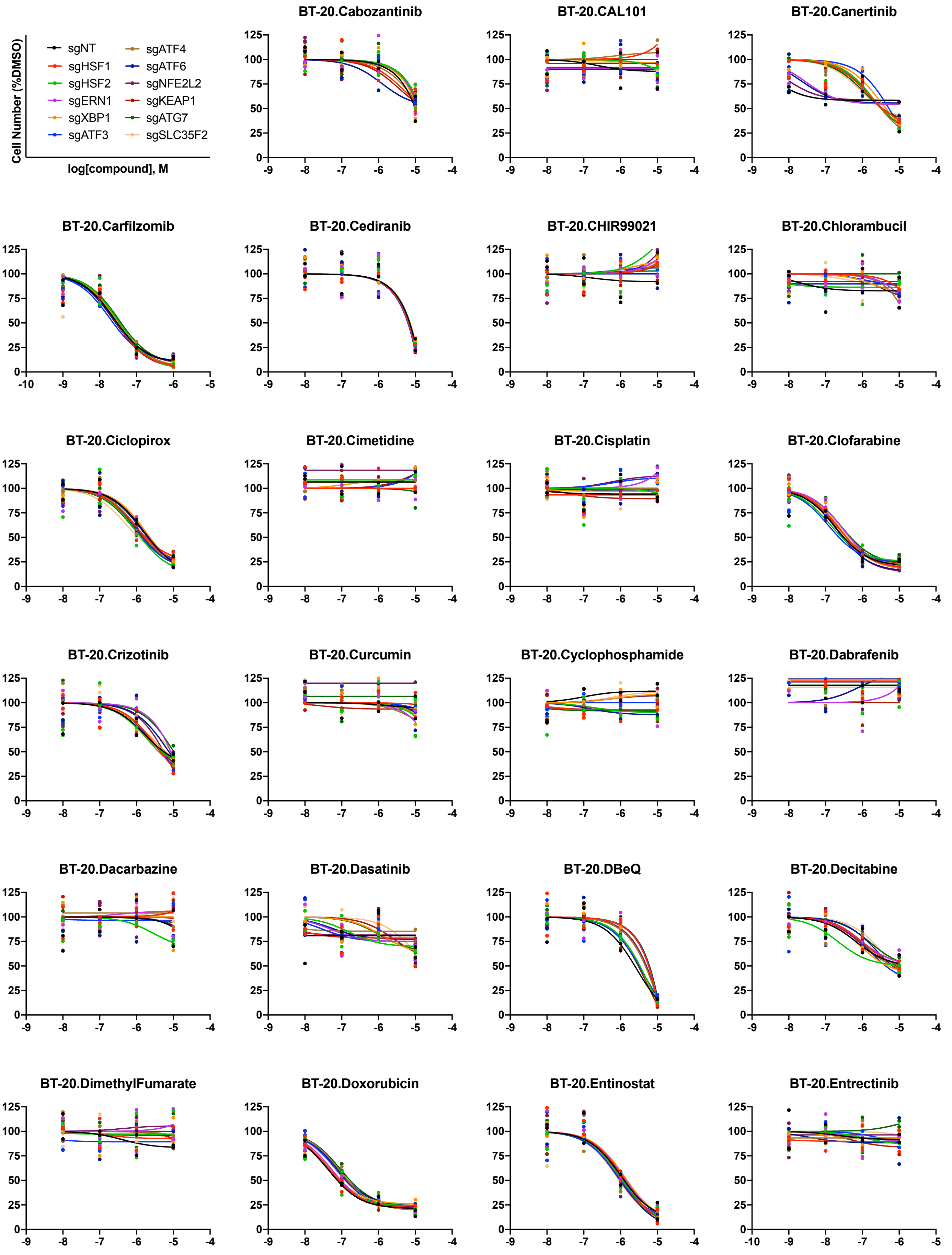

Supplementary Fig. 5 (continued). Dose-response curves generated using QMAP-Seq. Each plot represents a unique cell line-compound combination. Each data point represents one of two biologically independent replicates for the indicated sgRNA.

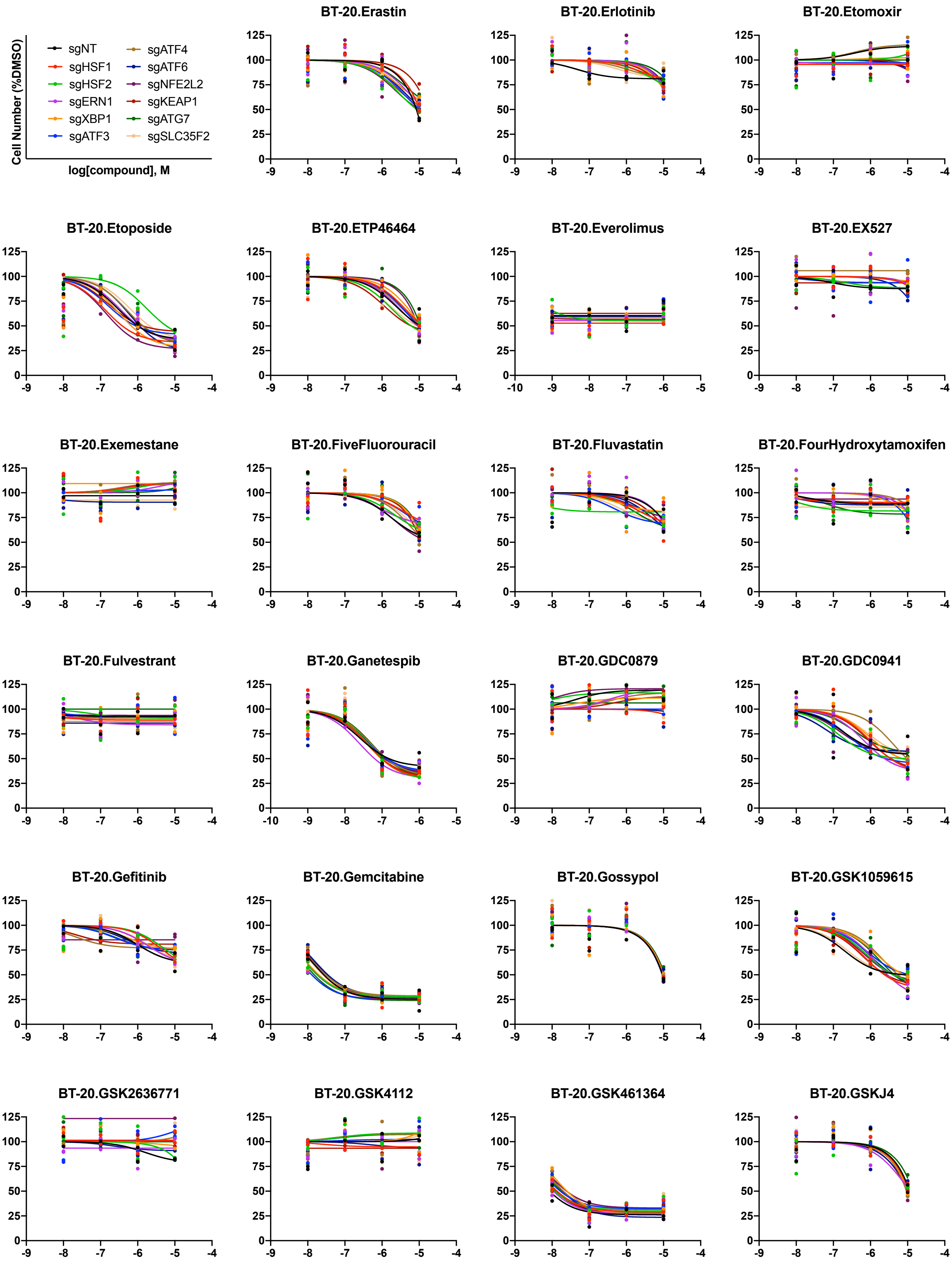

Supplementary Fig. 5 (continued). Dose-response curves generated using QMAP-Seq. Each plot represents a unique cell line-compound combination. Each data point represents one of two biologically independent replicates for the indicated sgRNA.

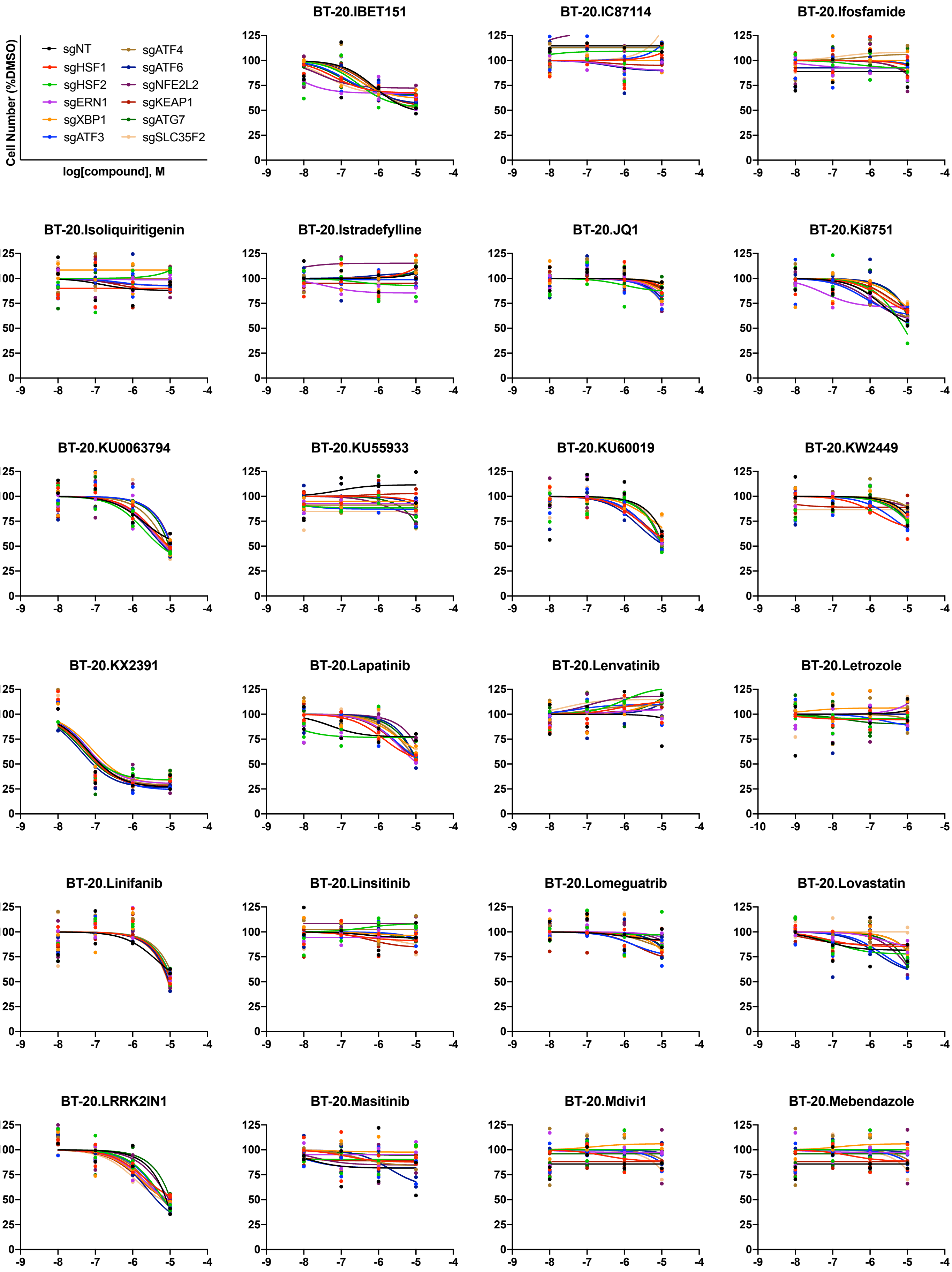

Supplementary Fig. 5 (continued). Dose-response curves generated using QMAP-Seq. Each plot represents a unique cell line-compound combination. Each data point represents one of two biologically independent replicates for the indicated sgRNA.

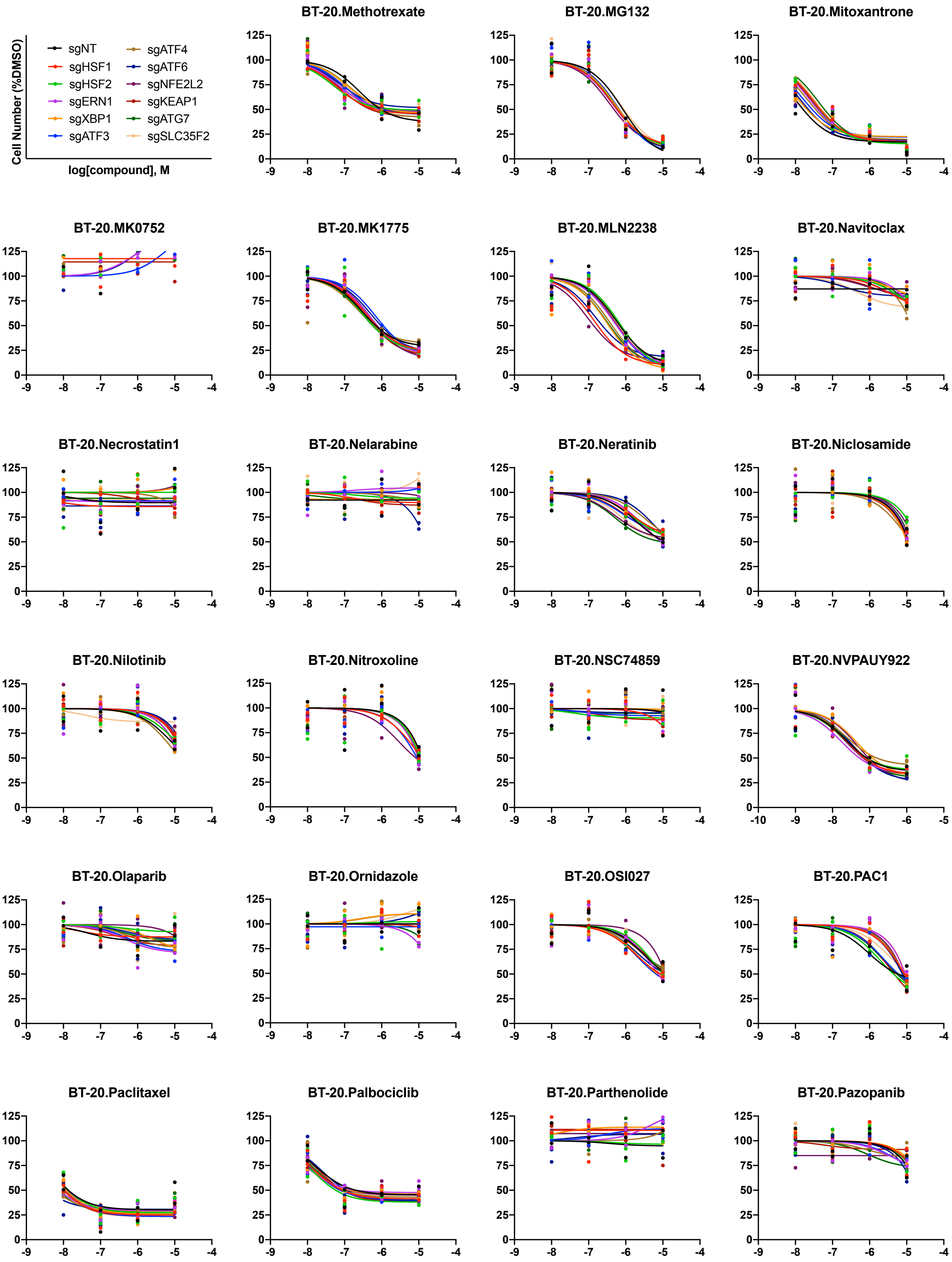

Supplementary Fig. 5 (continued). Dose-response curves generated using QMAP-Seq. Each plot represents a unique cell line-compound combination. Each data point represents one of two biologically independent replicates for the indicated sgRNA.

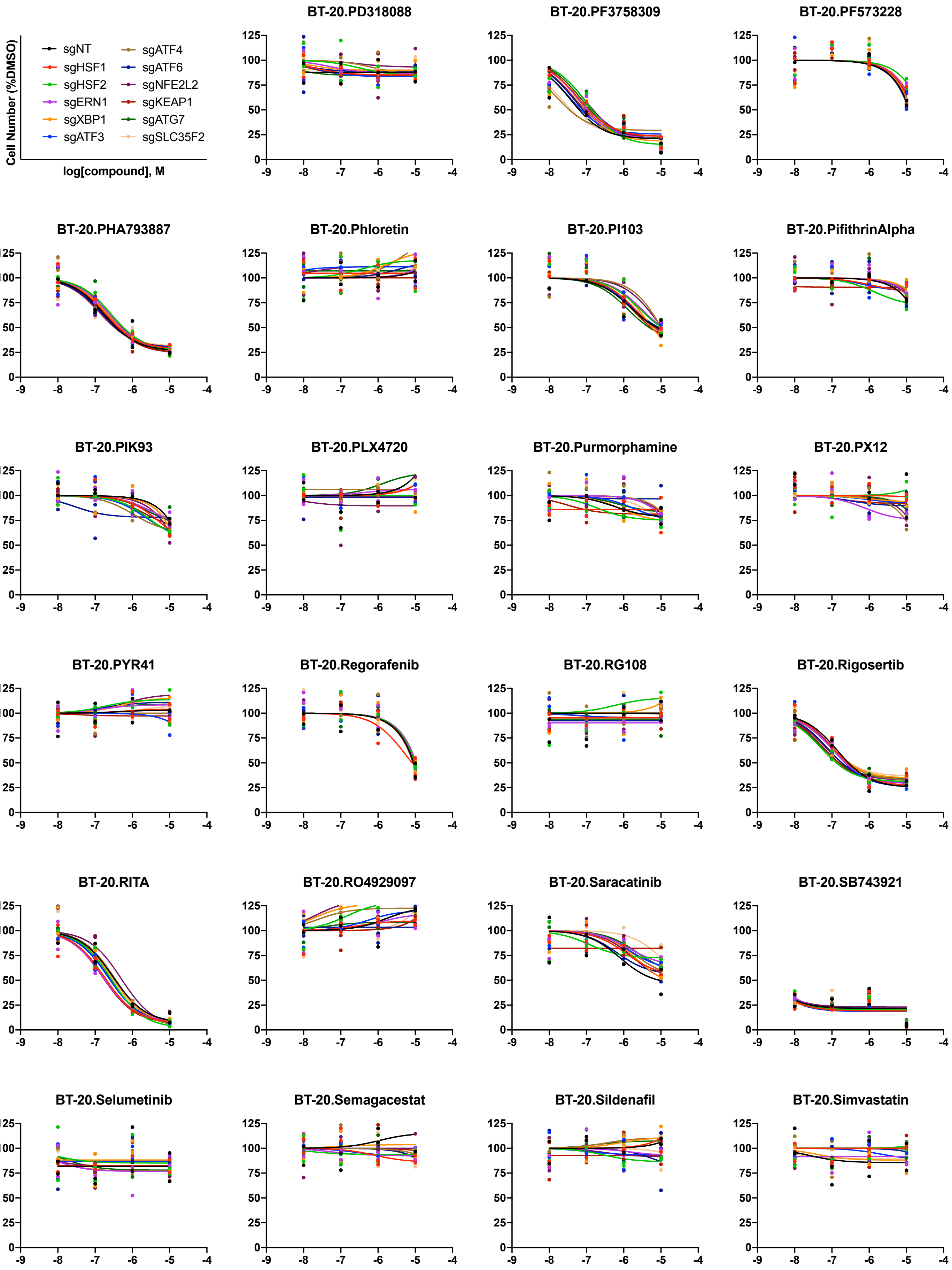

Supplementary Fig. 5 (continued). Dose-response curves generated using QMAP-Seq. Each plot represents a unique cell line-compound combination. Each data point represents one of two biologically independent replicates for the indicated sgRNA.

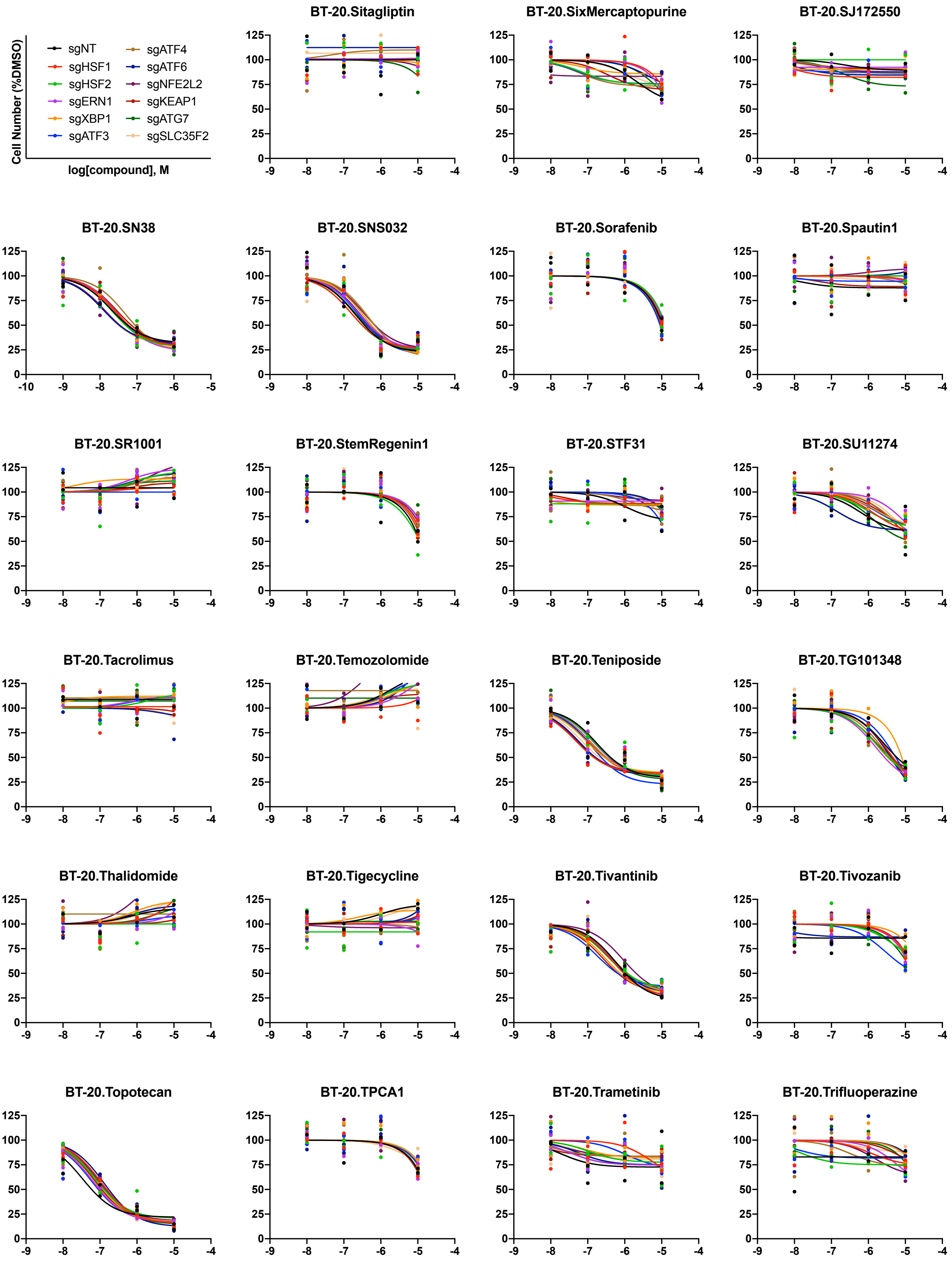

Supplementary Fig. 5 (continued). Dose-response curves generated using QMAP-Seq. Each plot represents a unique cell line-compound combination. Each data point represents one of two biologically independent replicates for the indicated sgRNA.

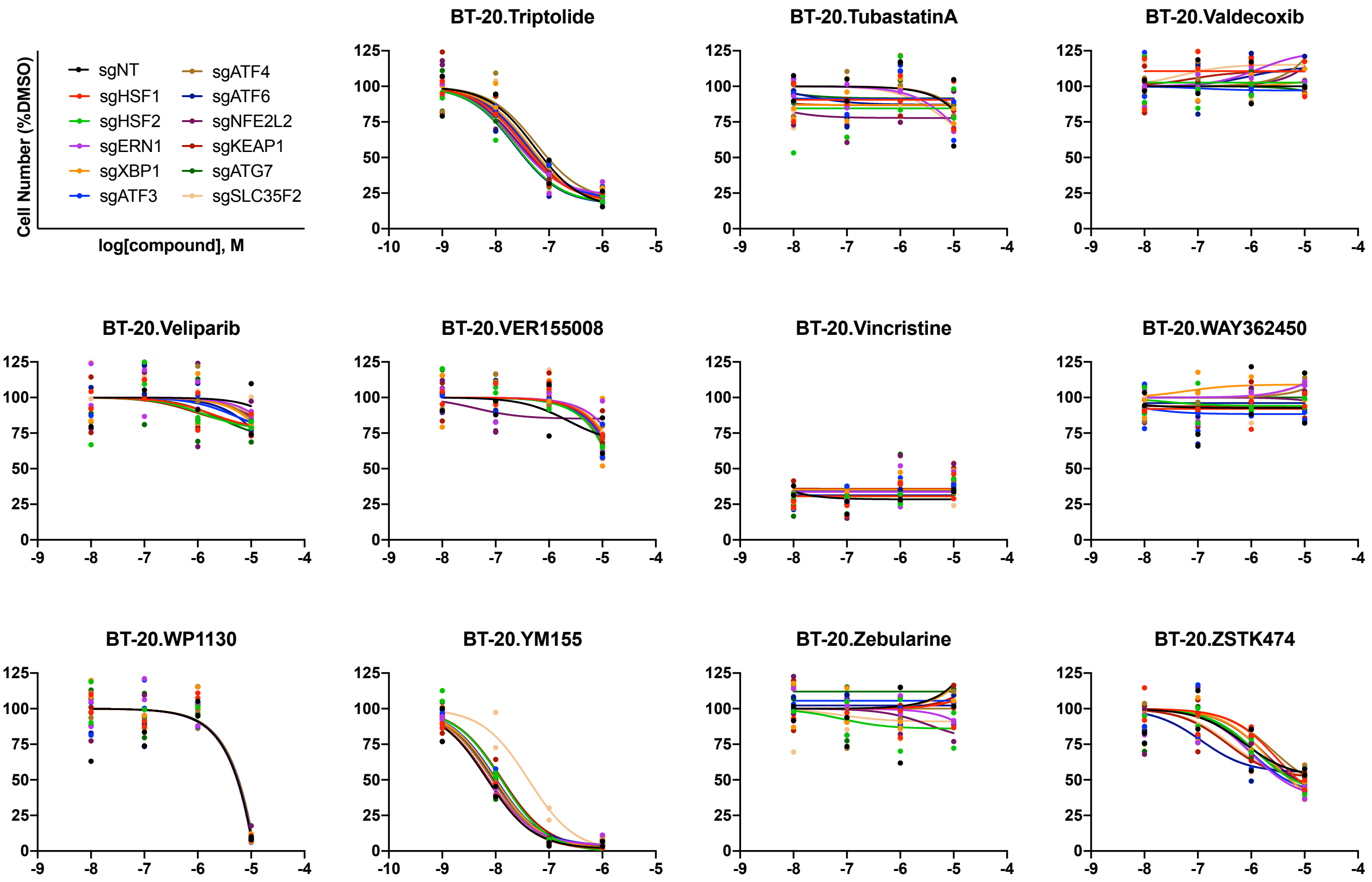

Supplementary Fig. 5 (continued). Dose-response curves generated using QMAP-Seq. Each plot represents a unique cell line-compound combination. Each data point represents one of two biologically independent replicates for the indicated sgRNA.

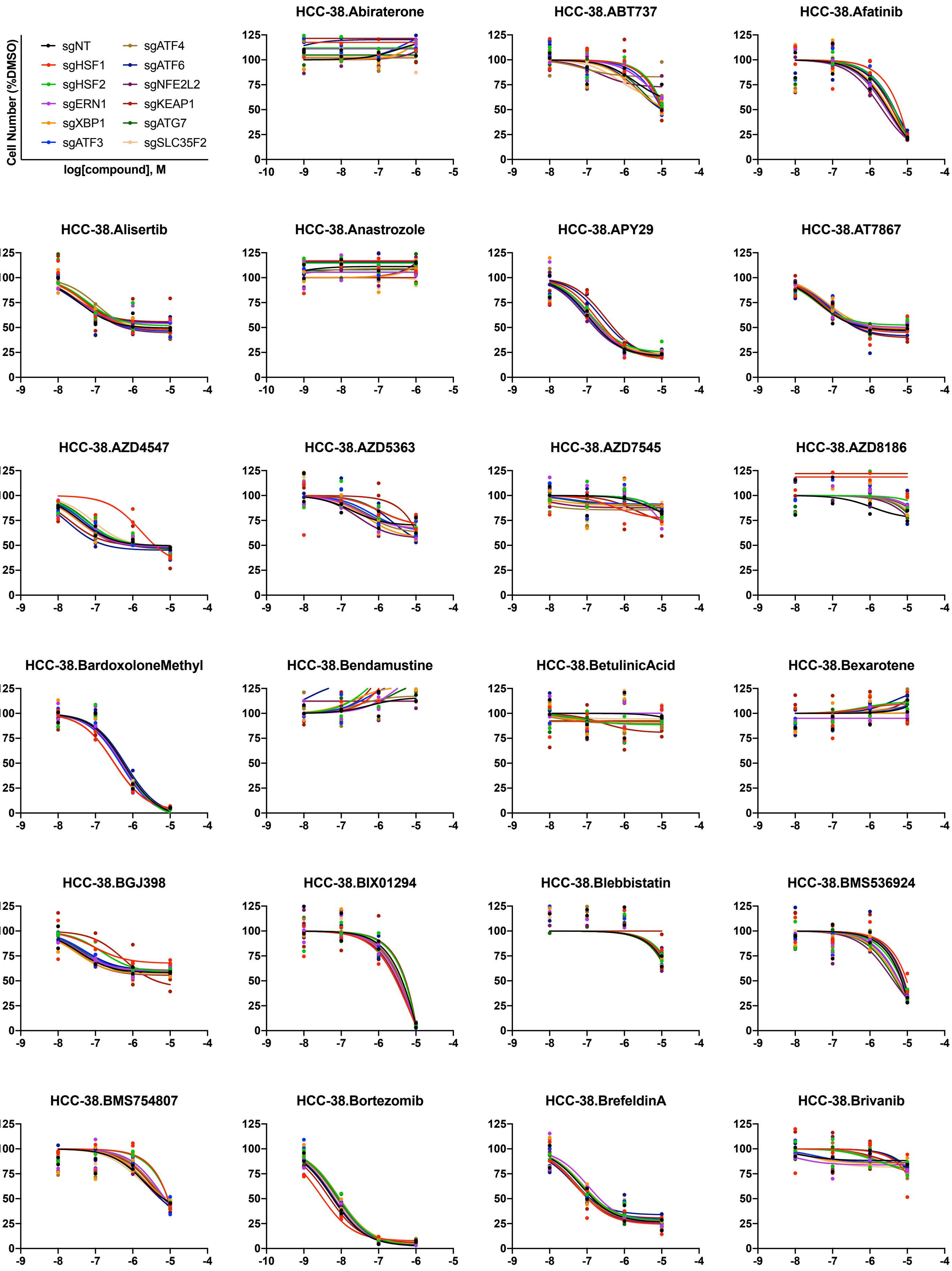

Supplementary Fig. 5 (continued). Dose-response curves generated using QMAP-Seq. Each plot represents a unique cell line-compound combination. Each data point represents one of two biologically independent replicates for the indicated sgRNA.

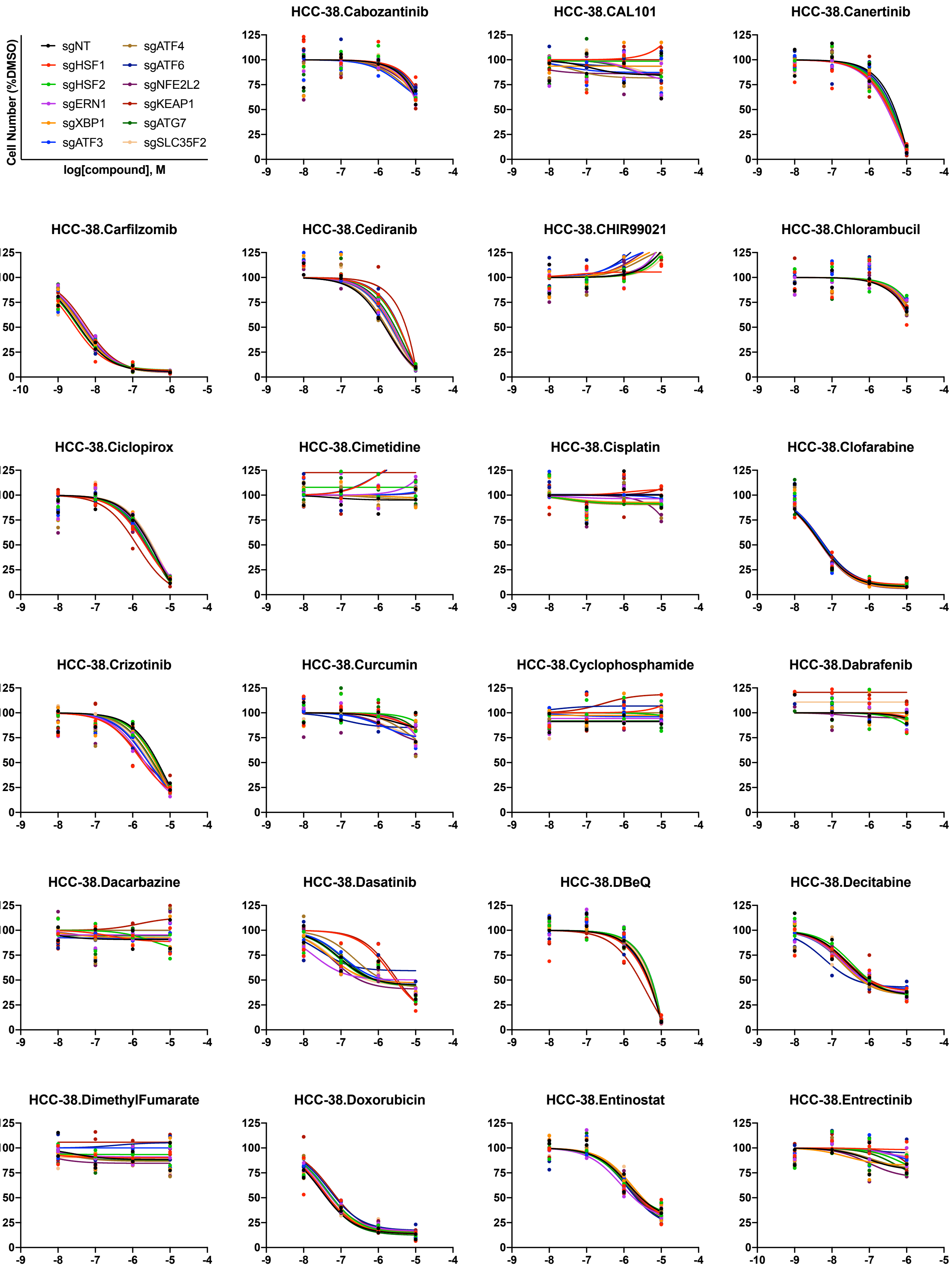

Supplementary Fig. 5 (continued). Dose-response curves generated using QMAP-Seq. Each plot represents a unique cell line-compound combination. Each data point represents one of two biologically independent replicates for the indicated sgRNA.

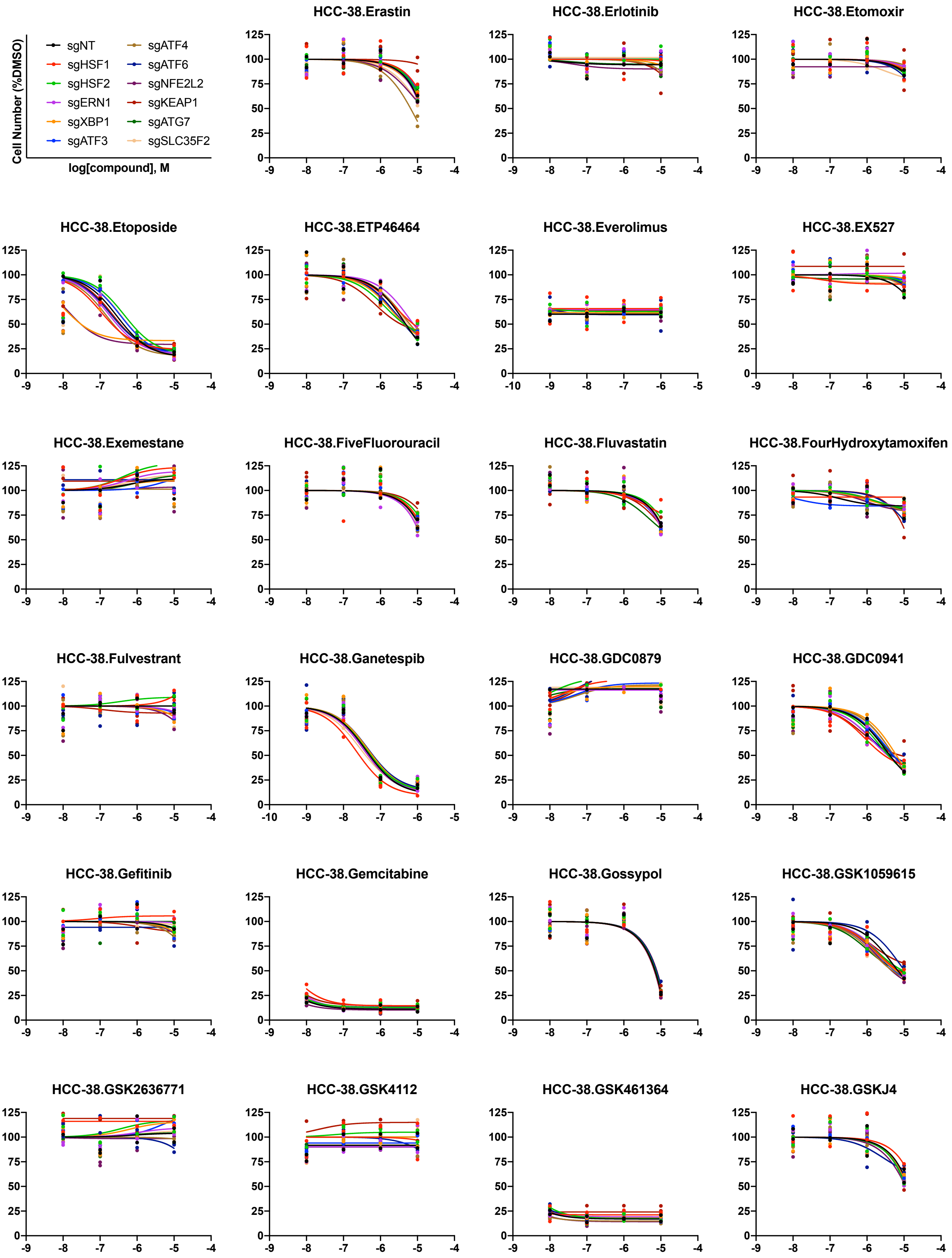

Supplementary Fig. 5 (continued). Dose-response curves generated using QMAP-Seq. Each plot represents a unique cell line-compound combination. Each data point represents one of two biologically independent replicates for the indicated sgRNA.

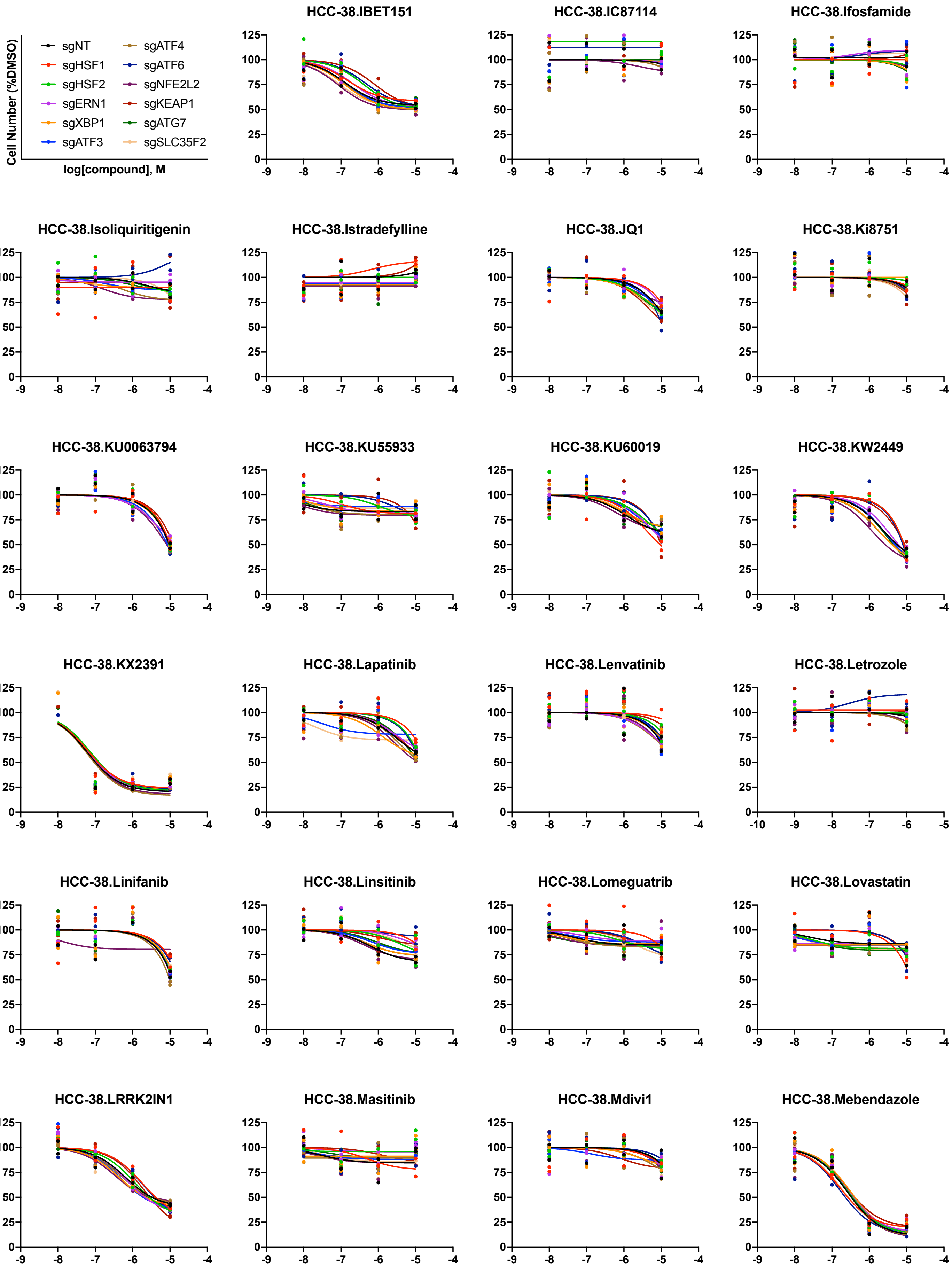

Supplementary Fig. 5 (continued). Dose-response curves generated using QMAP-Seq. Each plot represents a unique cell line-compound combination. Each data point represents one of two biologically independent replicates for the indicated sgRNA.

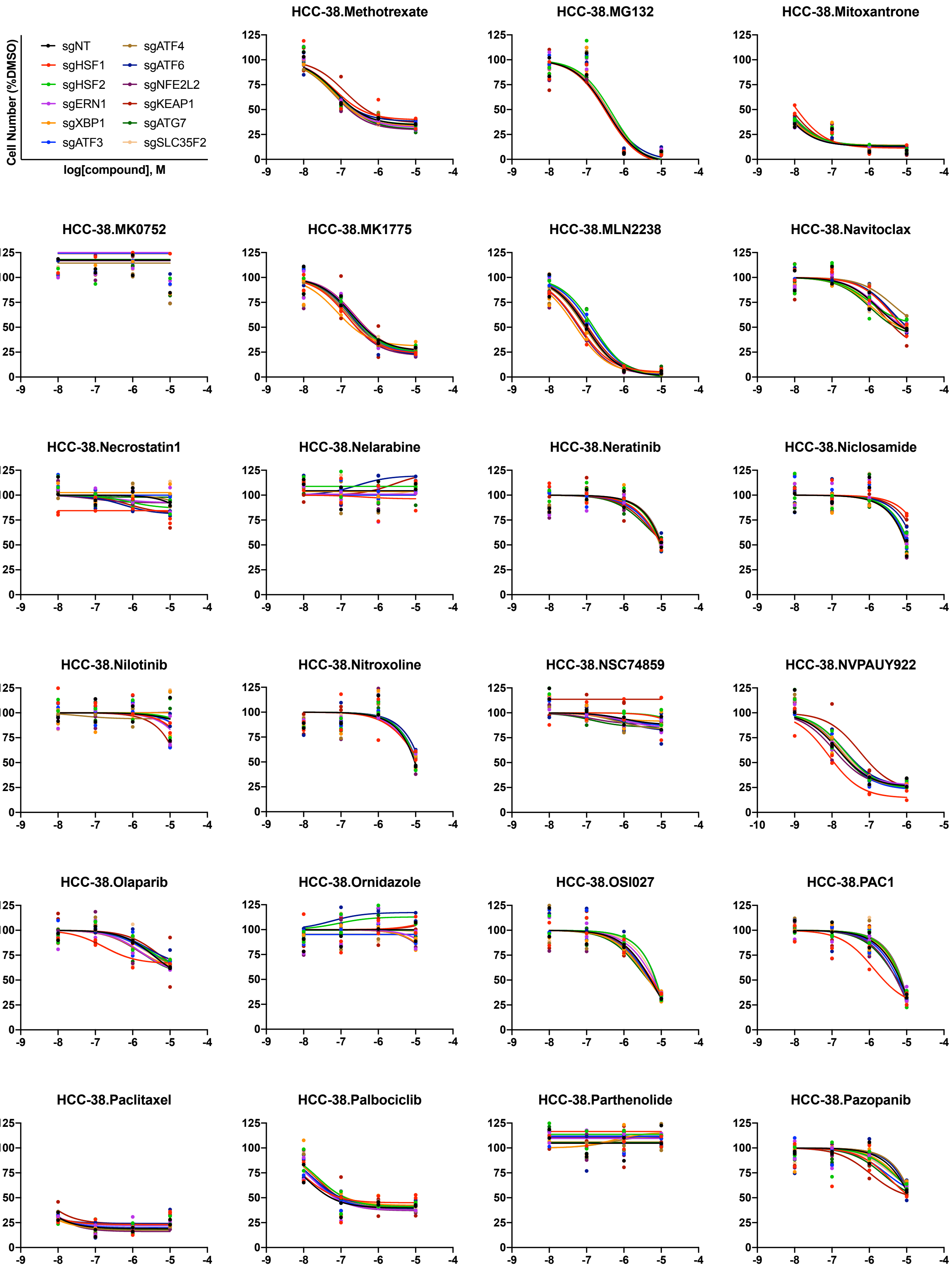

Supplementary Fig. 5 (continued). Dose-response curves generated using QMAP-Seq. Each plot represents a unique cell line-compound combination. Each data point represents one of two biologically independent replicates for the indicated sgRNA.

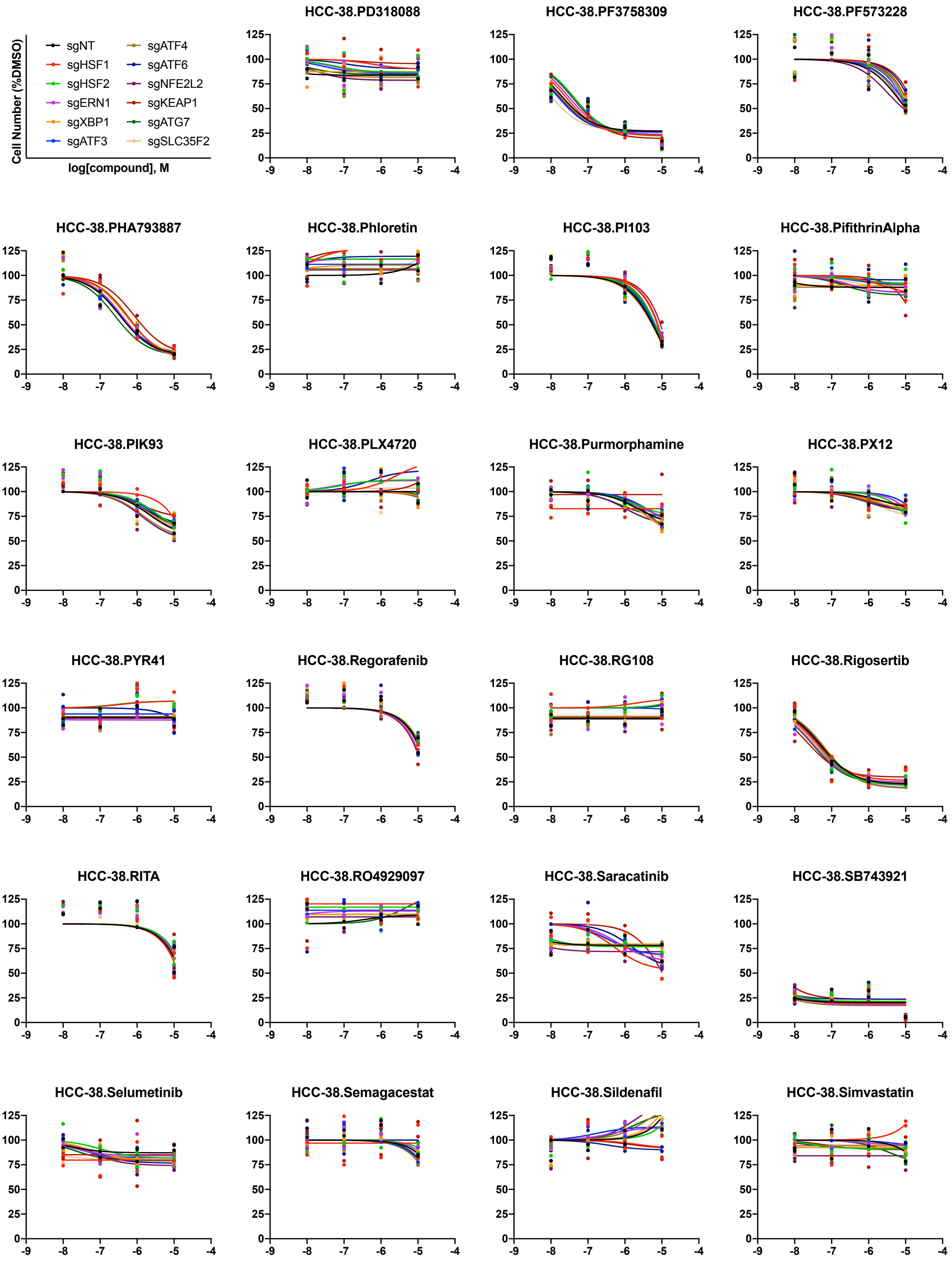

Supplementary Fig. 5 (continued). Dose-response curves generated using QMAP-Seq. Each plot represents a unique cell line-compound combination. Each data point represents one of two biologically independent replicates for the indicated sgRNA.

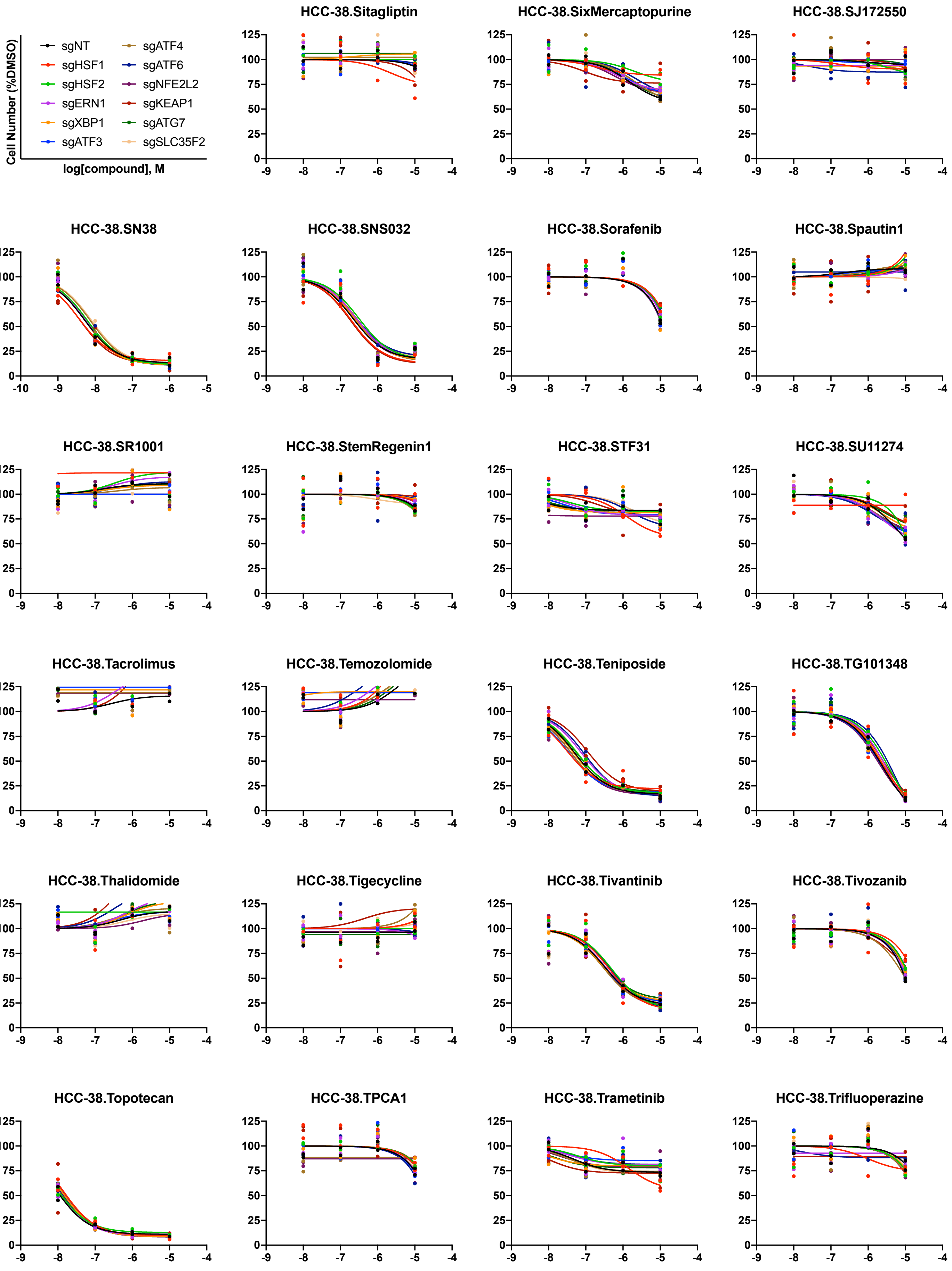

Supplementary Fig. 5 (continued). Dose-response curves generated using QMAP-Seq. Each plot represents a unique cell line-compound combination. Each data point represents one of two biologically independent replicates for the indicated sgRNA.

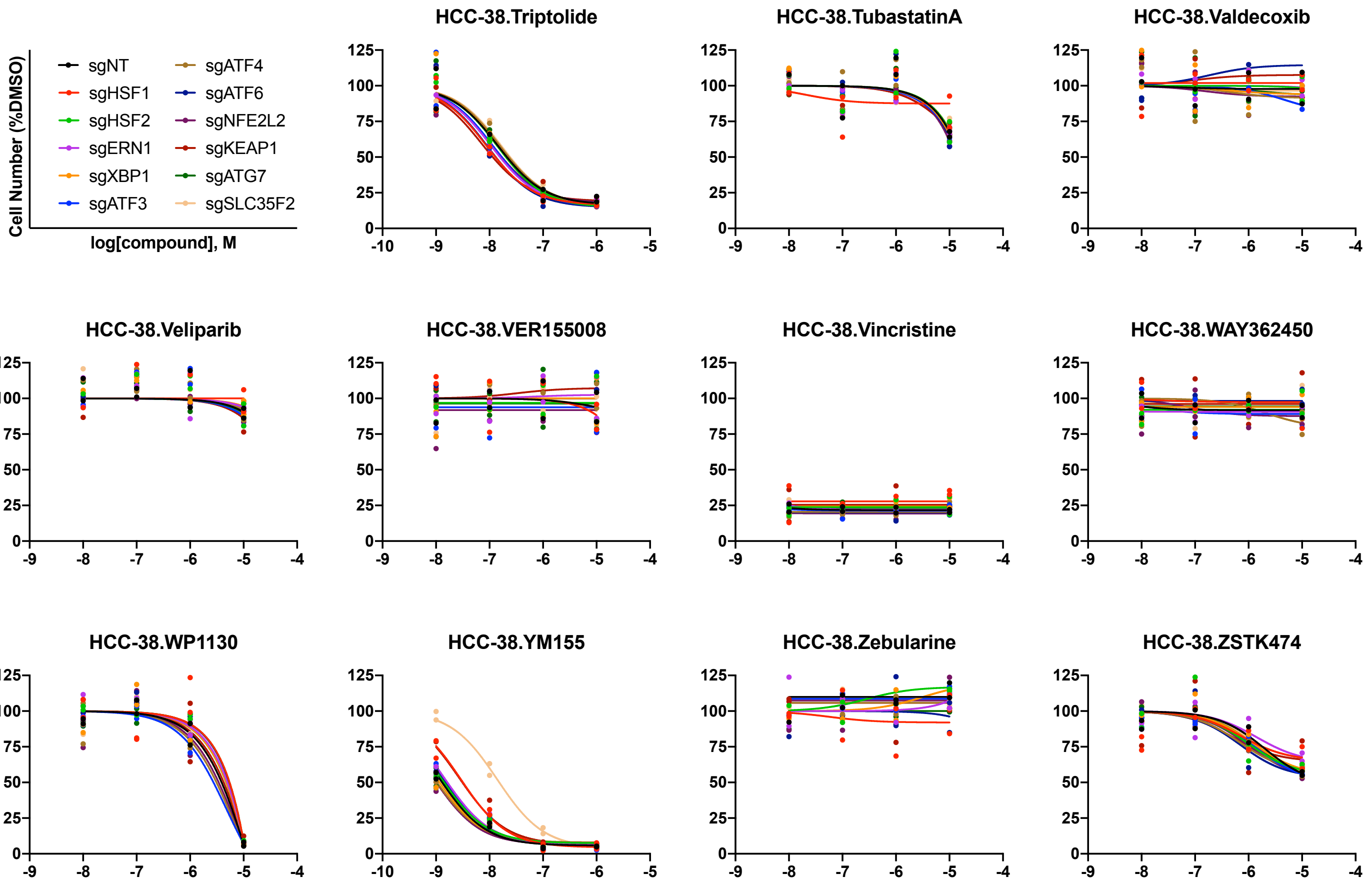

Supplementary Fig. 5 (continued). Dose-response curves generated using QMAP-Seq. Each plot represents a unique cell line-compound combination. Each data point represents one of two biologically independent replicates for the indicated sgRNA.

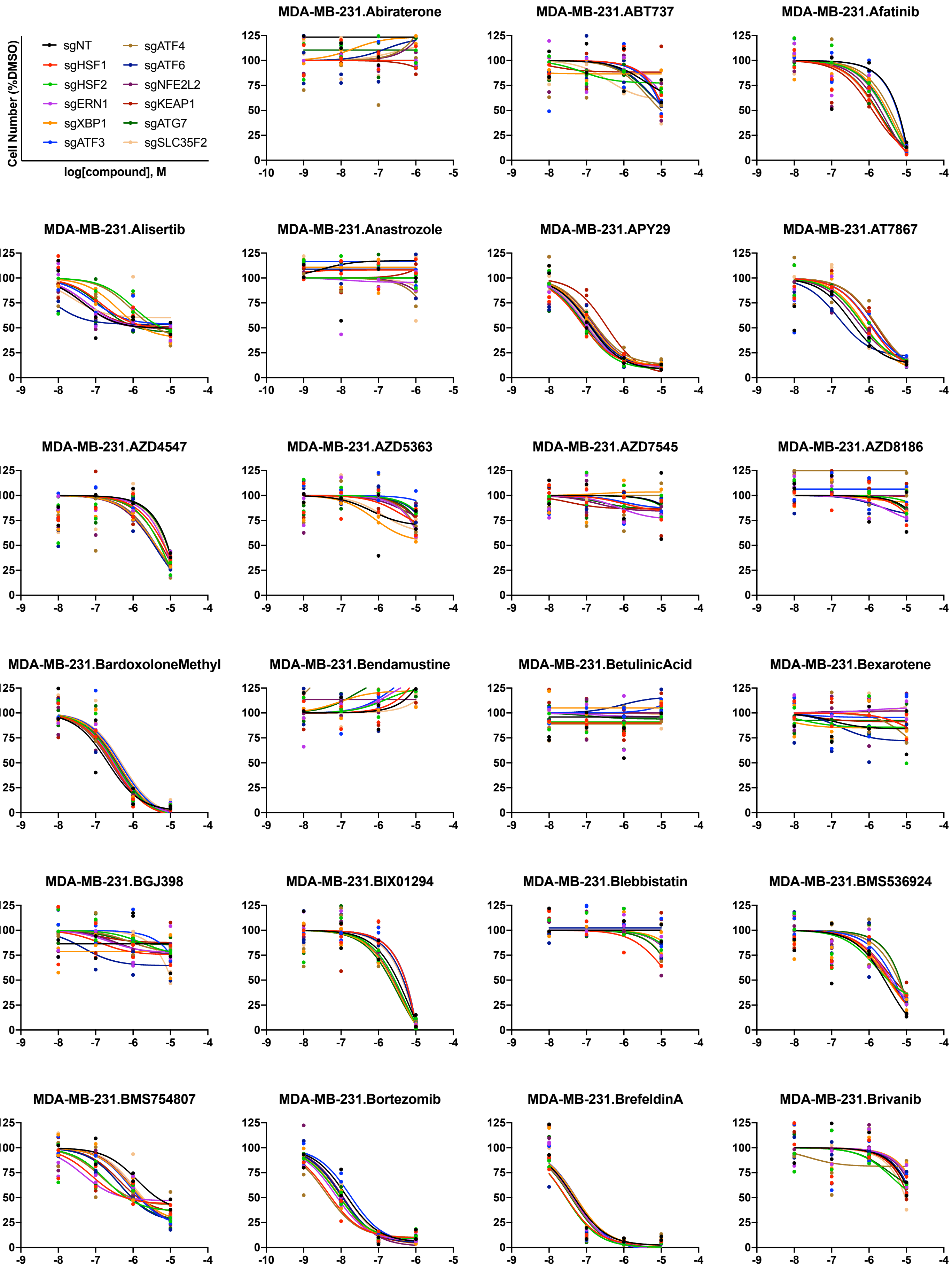

Supplementary Fig. 5 (continued). Dose-response curves generated using QMAP-Seq. Each plot represents a unique cell line-compound combination. Each data point represents one of two biologically independent replicates for the indicated sgRNA.

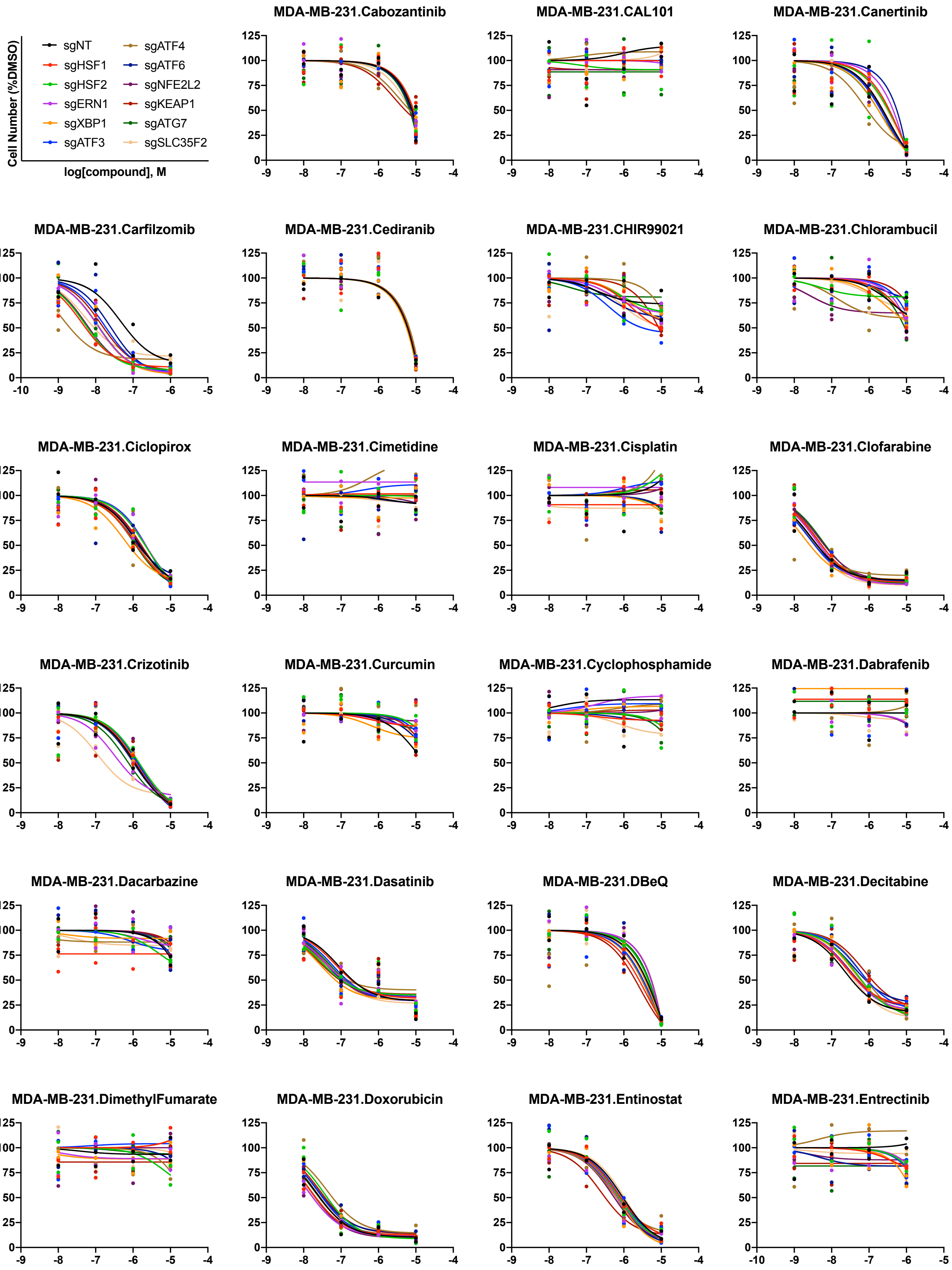

Supplementary Fig. 5 (continued). Dose-response curves generated using QMAP-Seq. Each plot represents a unique cell line-compound combination. Each data point represents one of two biologically independent replicates for the indicated sgRNA.

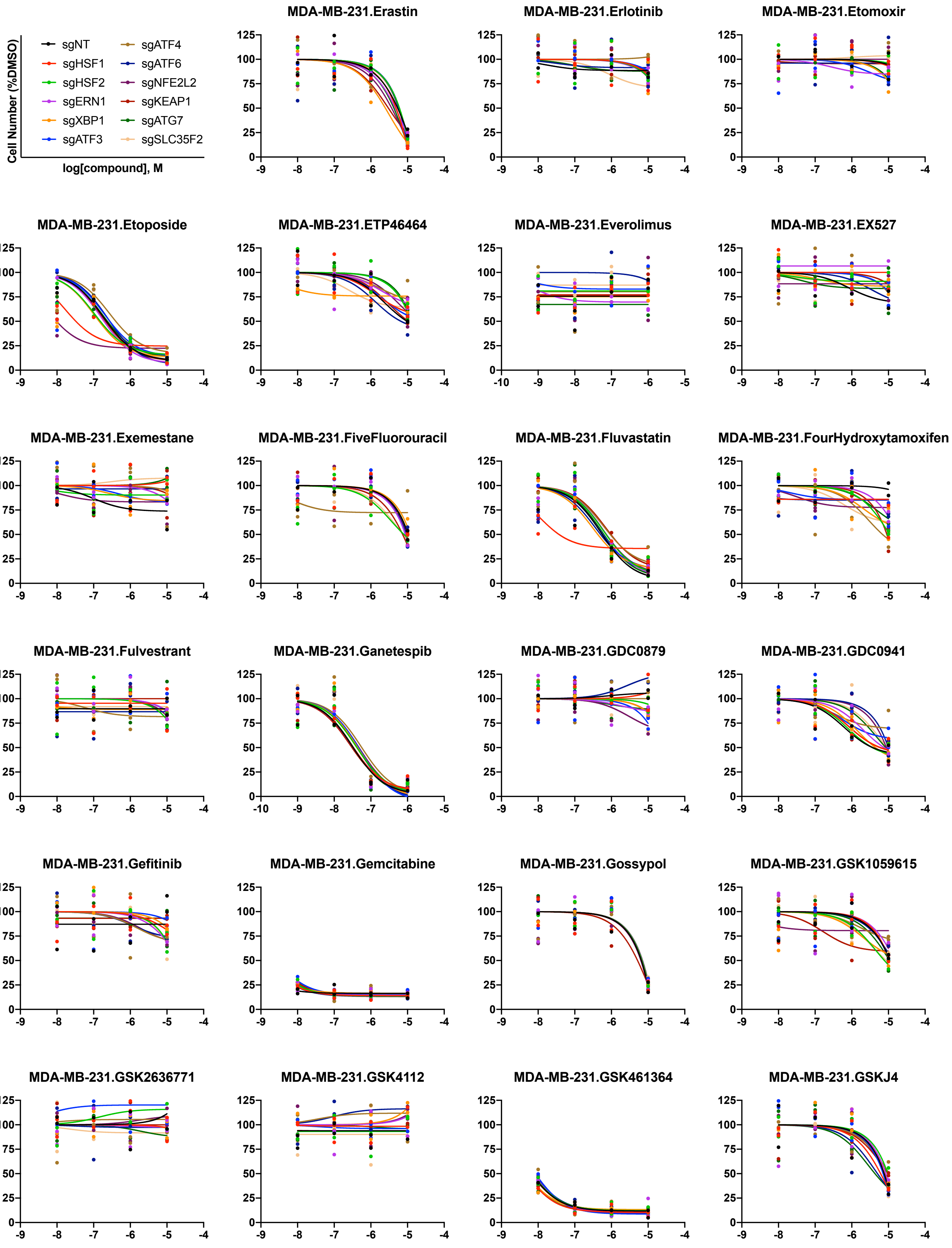

Supplementary Fig. 5 (continued). Dose-response curves generated using QMAP-Seq. Each plot represents a unique cell line-compound combination. Each data point represents one of two biologically independent replicates for the indicated sgRNA.

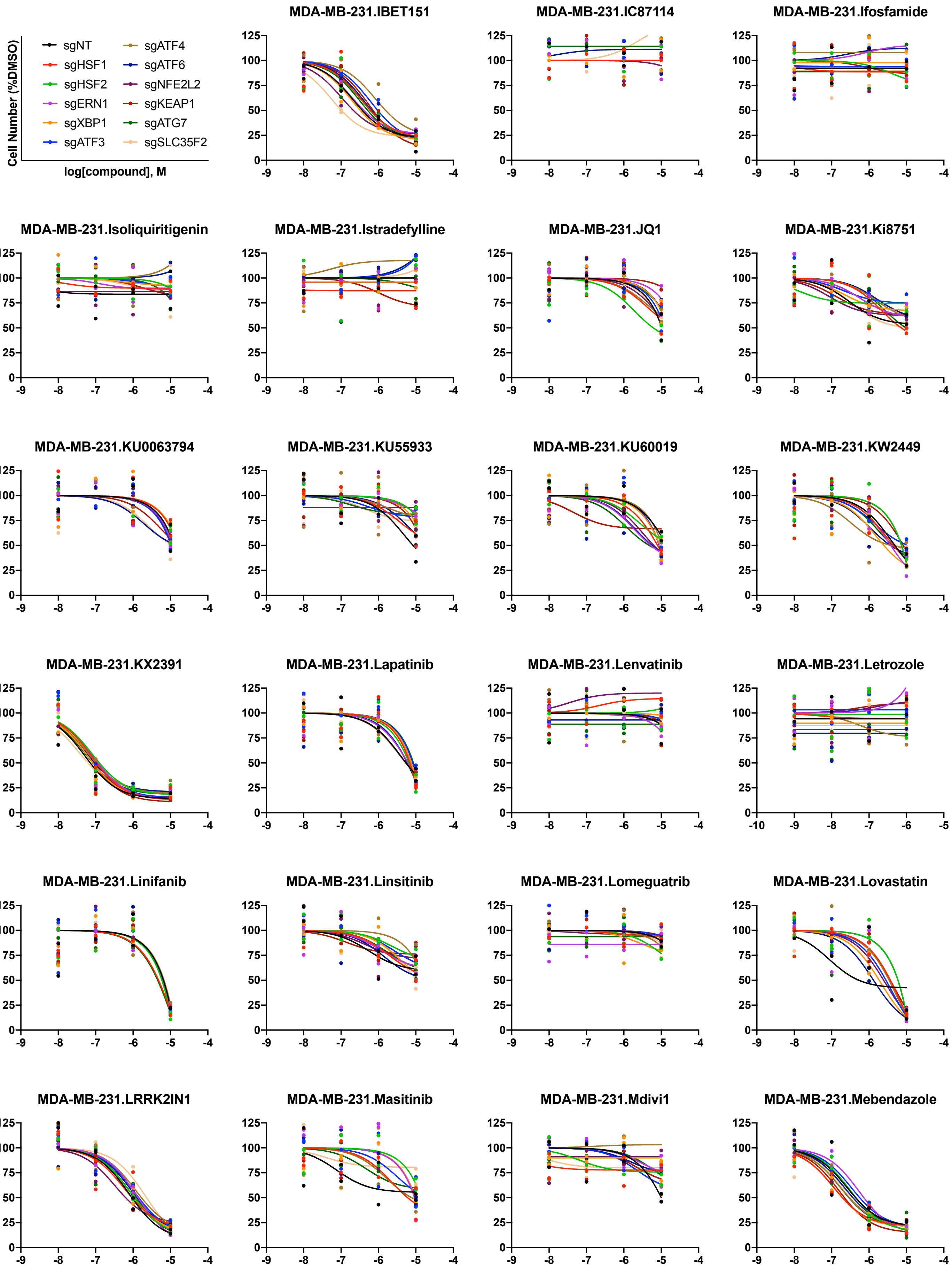

Supplementary Fig. 5 (continued). Dose-response curves generated using QMAP-Seq. Each plot represents a unique cell line-compound combination. Each data point represents one of two biologically independent replicates for the indicated sgRNA.

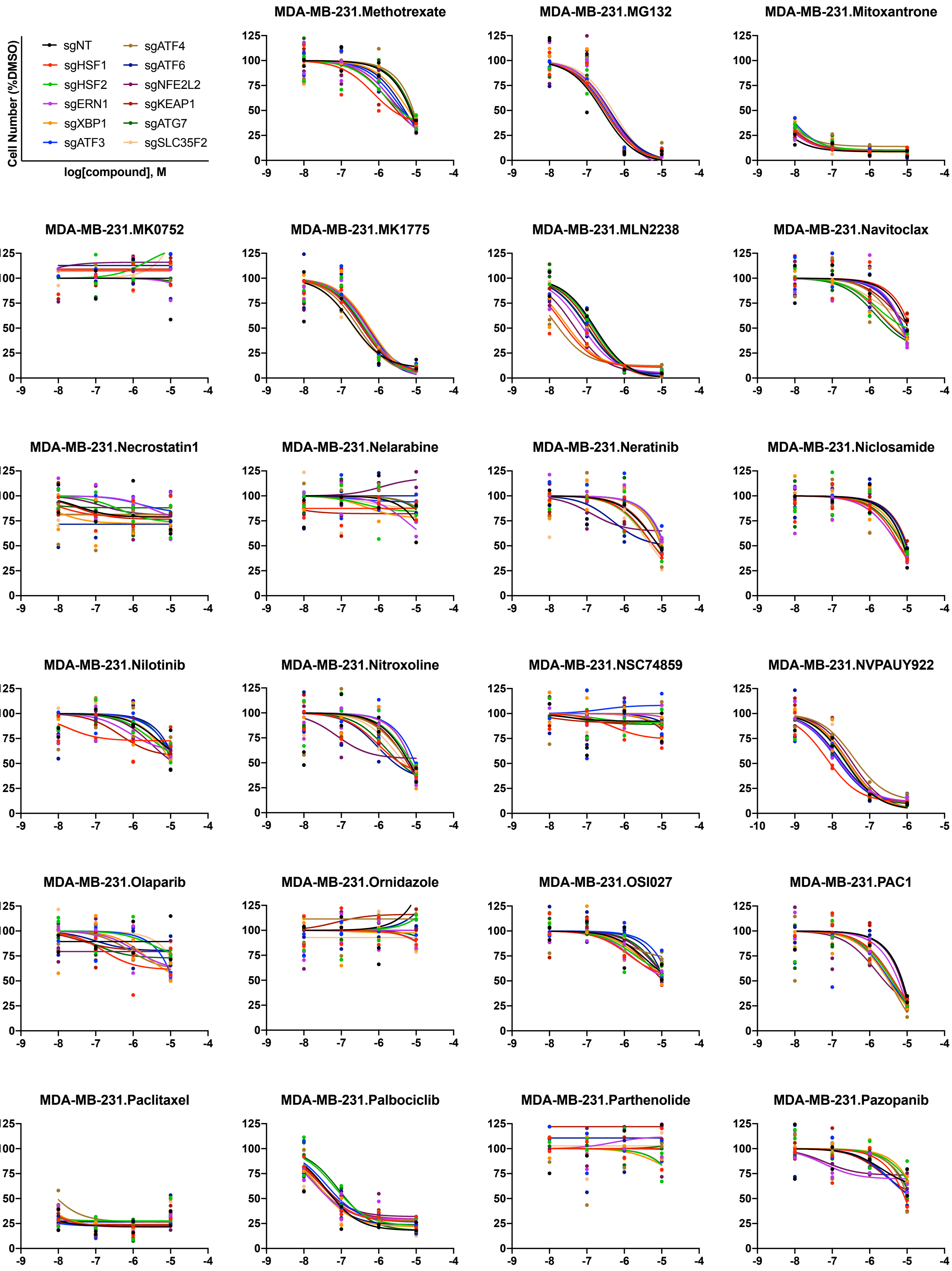

Supplementary Fig. 5 (continued). Dose-response curves generated using QMAP-Seq. Each plot represents a unique cell line-compound combination. Each data point represents one of two biologically independent replicates for the indicated sgRNA.

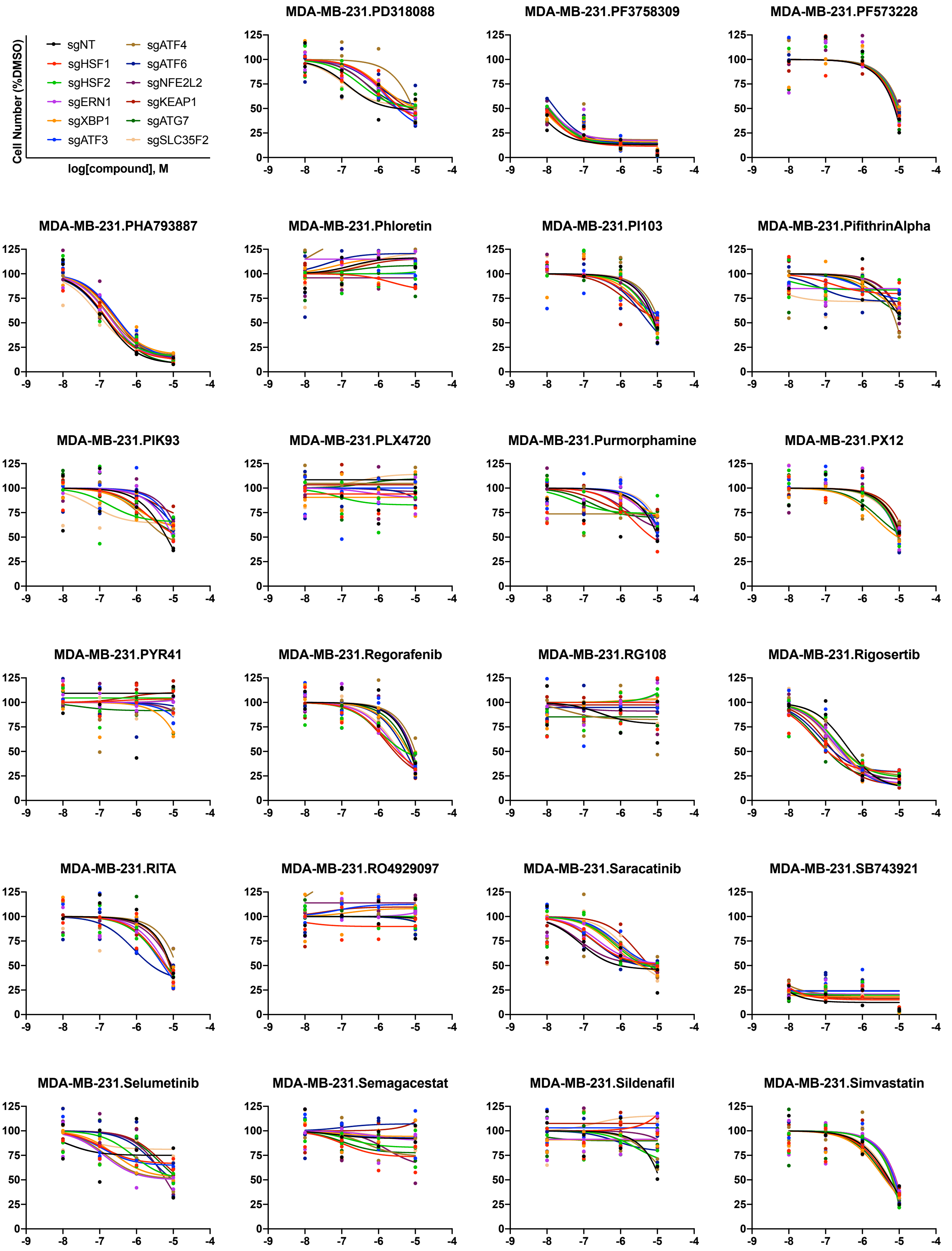

Supplementary Fig. 5 (continued). Dose-response curves generated using QMAP-Seq. Each plot represents a unique cell line-compound combination. Each data point represents one of two biologically independent replicates for the indicated sgRNA.

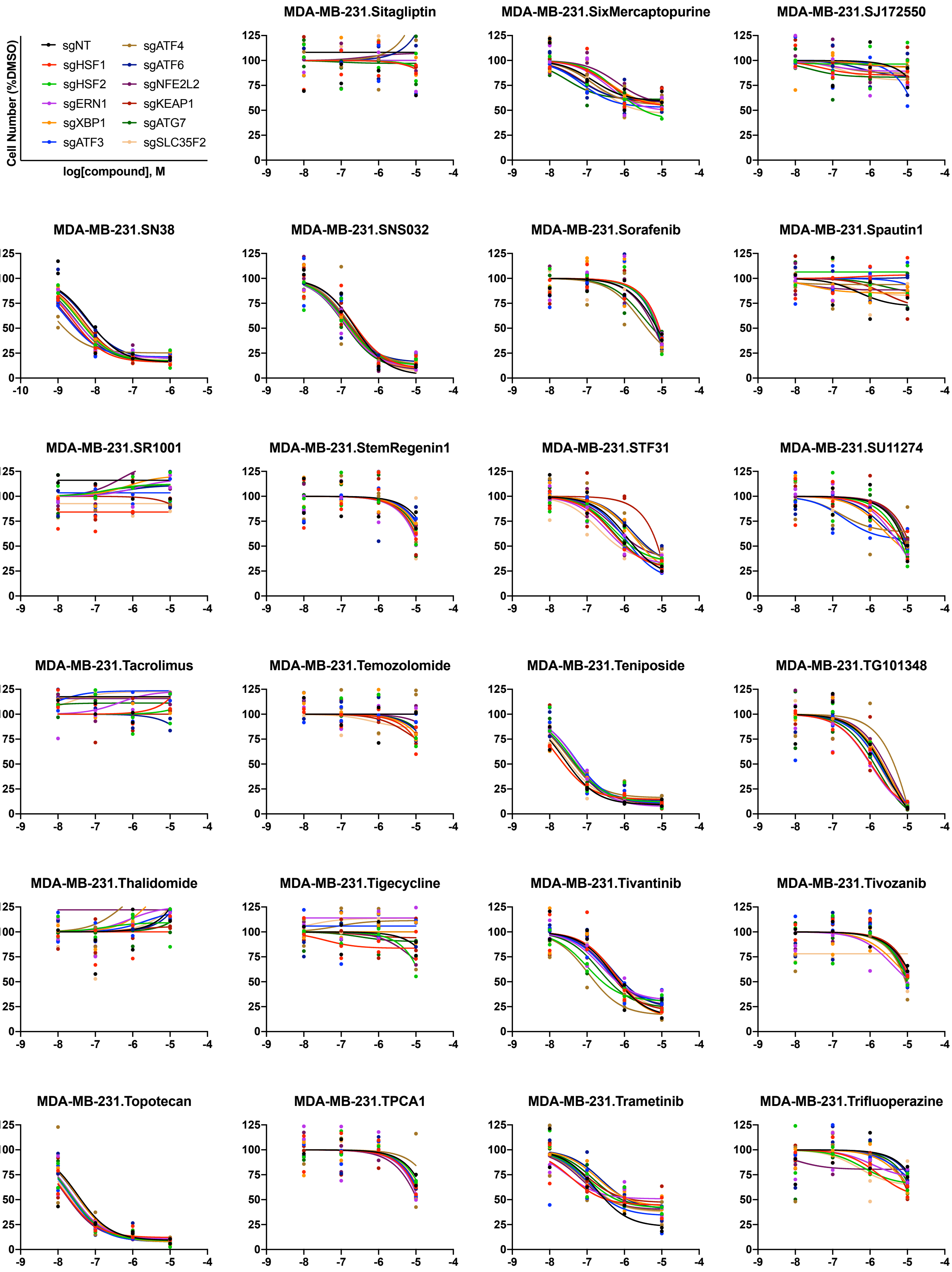

Supplementary Fig. 5 (continued). Dose-response curves generated using QMAP-Seq. Each plot represents a unique cell line-compound combination. Each data point represents one of two biologically independent replicates for the indicated sgRNA.

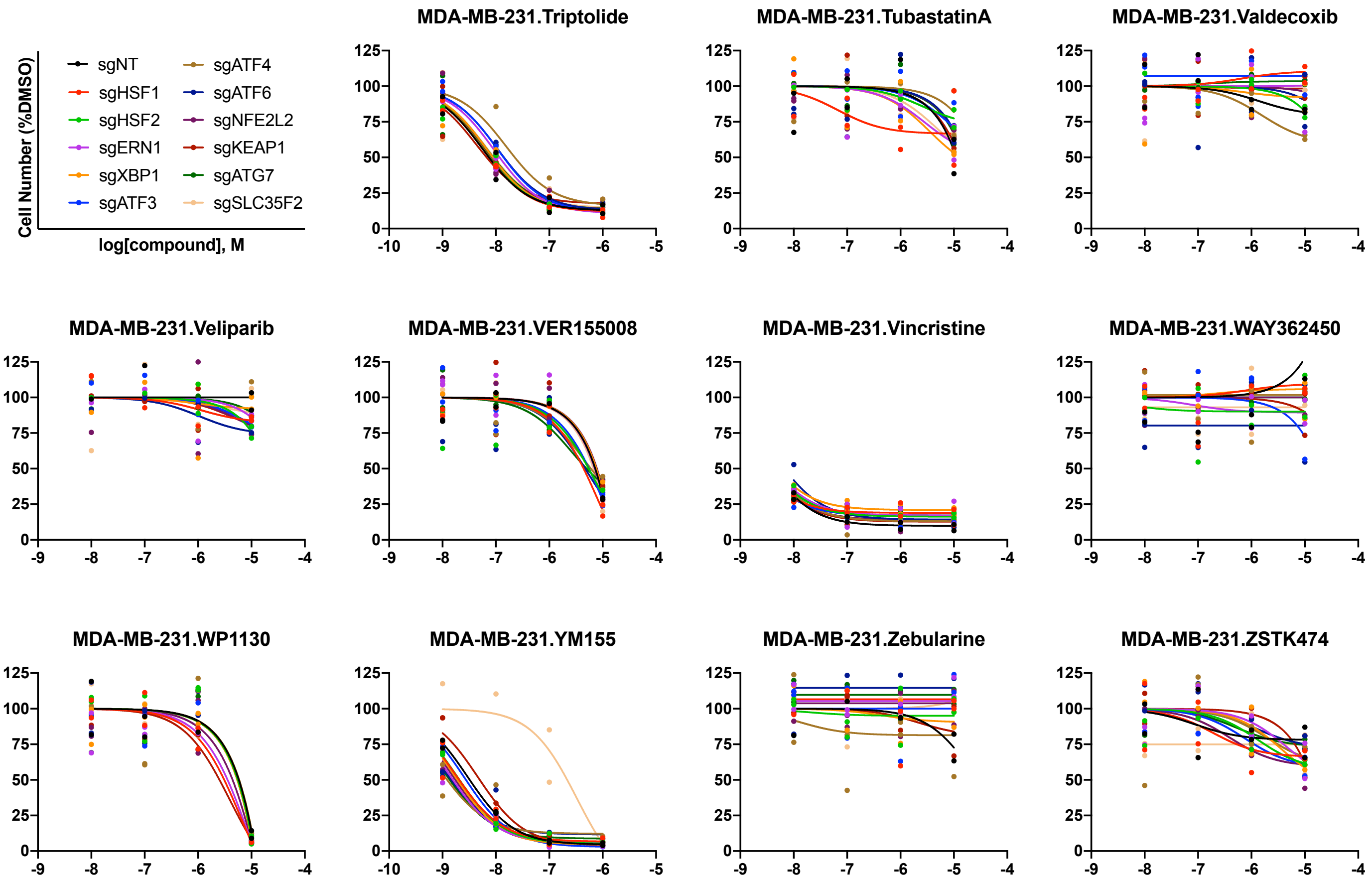

Supplementary Fig. 5 (continued). Dose-response curves generated using QMAP-Seq. Each plot represents a unique cell line-compound combination. Each data point represents one of two biologically independent replicates for the indicated sgRNA.

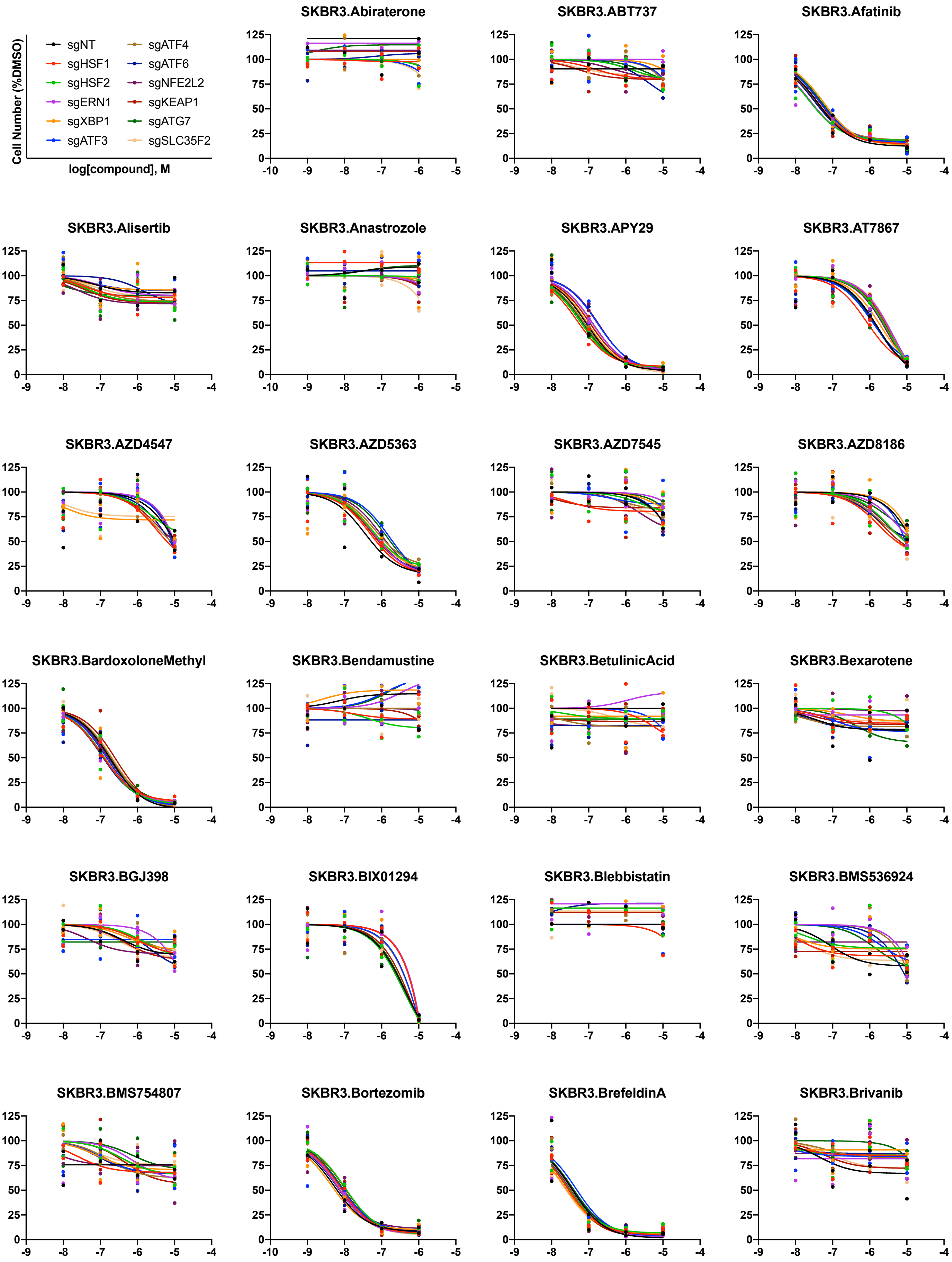

Supplementary Fig. 5 (continued). Dose-response curves generated using QMAP-Seq. Each plot represents a unique cell line-compound combination. Each data point represents one of two biologically independent replicates for the indicated sgRNA.

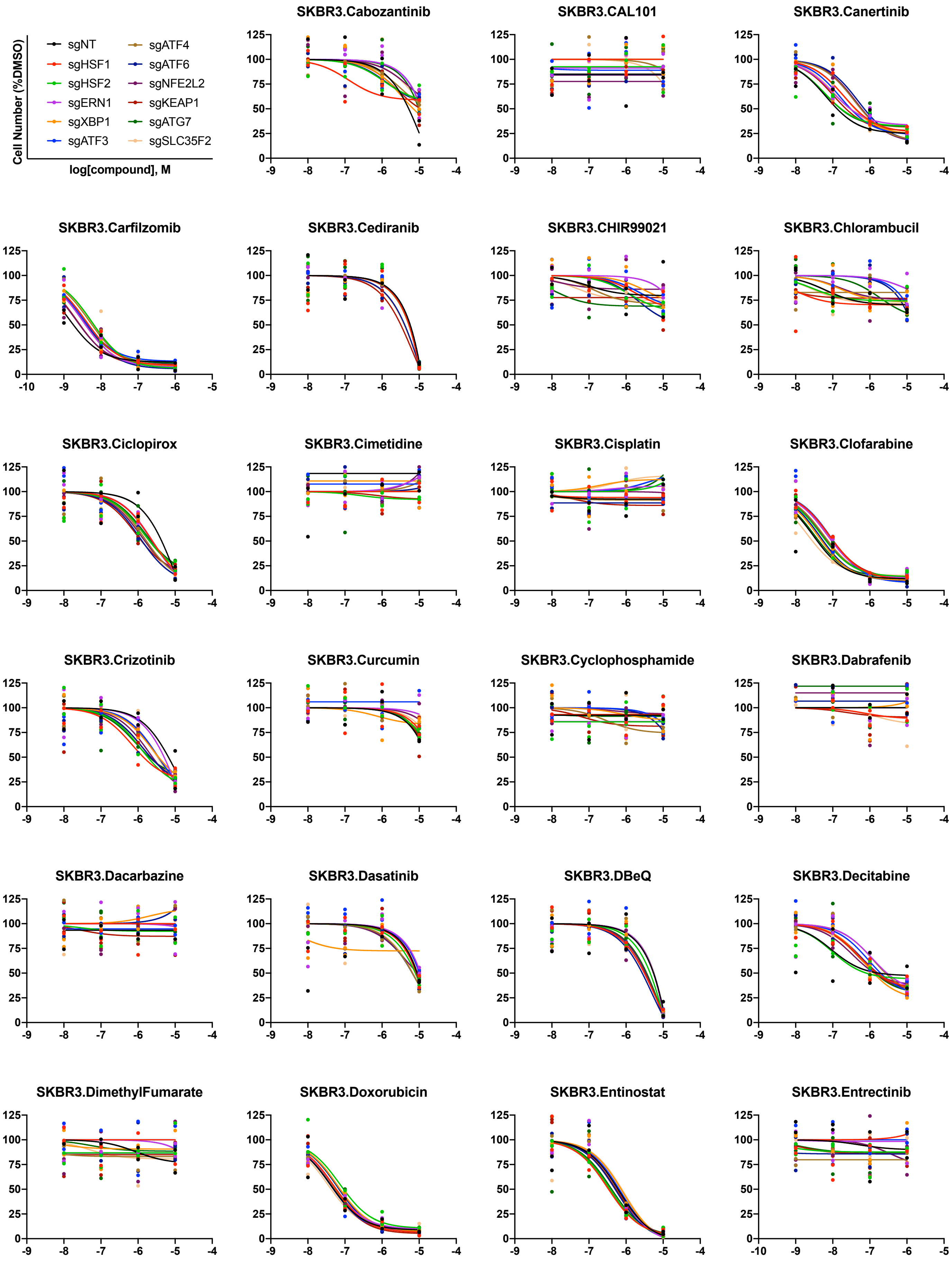

Supplementary Fig. 5 (continued). Dose-response curves generated using QMAP-Seq. Each plot represents a unique cell line-compound combination. Each data point represents one of two biologically independent replicates for the indicated sgRNA.

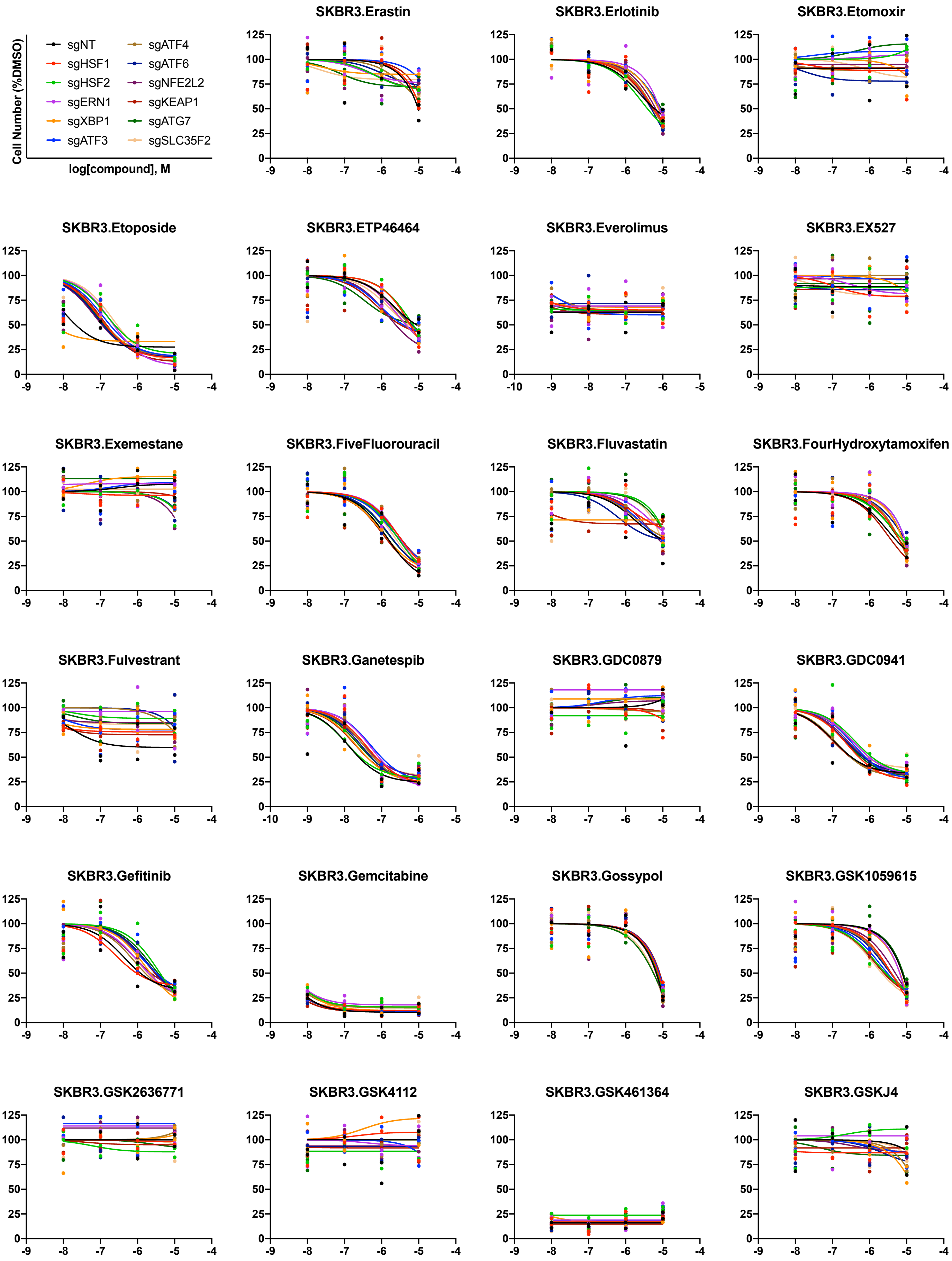

Supplementary Fig. 5 (continued). Dose-response curves generated using QMAP-Seq. Each plot represents a unique cell line-compound combination. Each data point represents one of two biologically independent replicates for the indicated sgRNA.

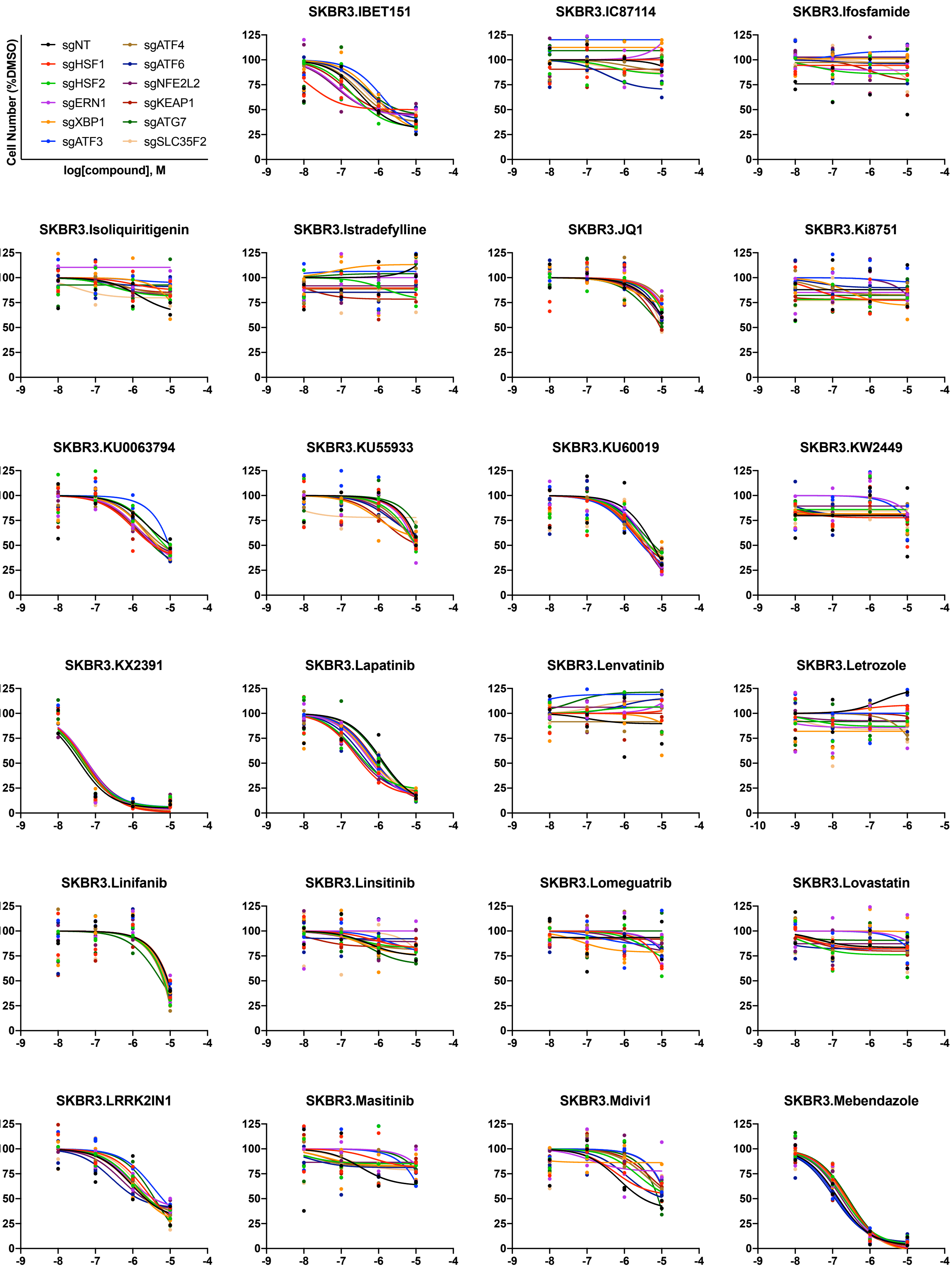

Supplementary Fig. 5 (continued). Dose-response curves generated using QMAP-Seq. Each plot represents a unique cell line-compound combination. Each data point represents one of two biologically independent replicates for the indicated sgRNA.

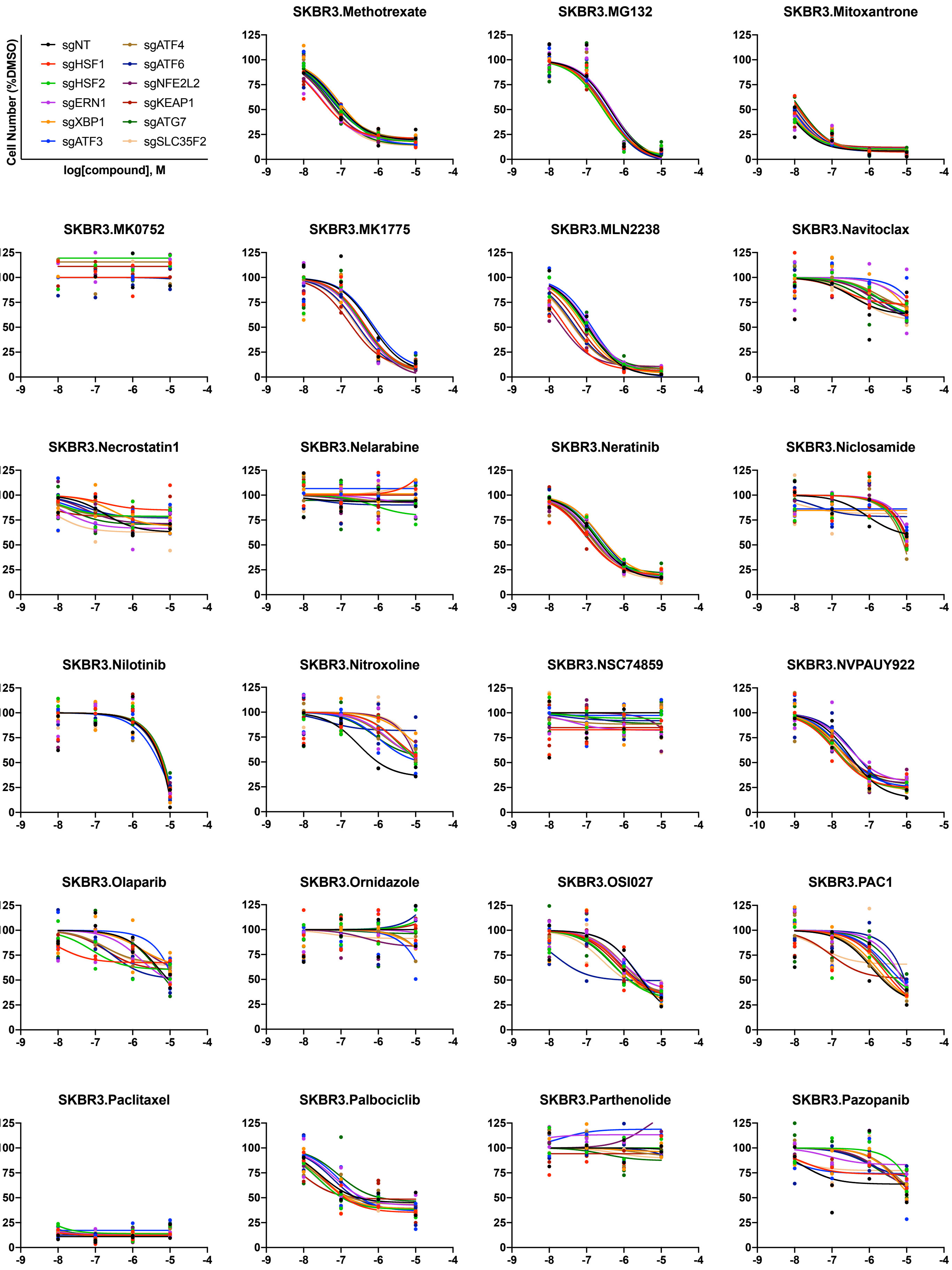

Supplementary Fig. 5 (continued). Dose-response curves generated using QMAP-Seq. Each plot represents a unique cell line-compound combination. Each data point represents one of two biologically independent replicates for the indicated sgRNA.

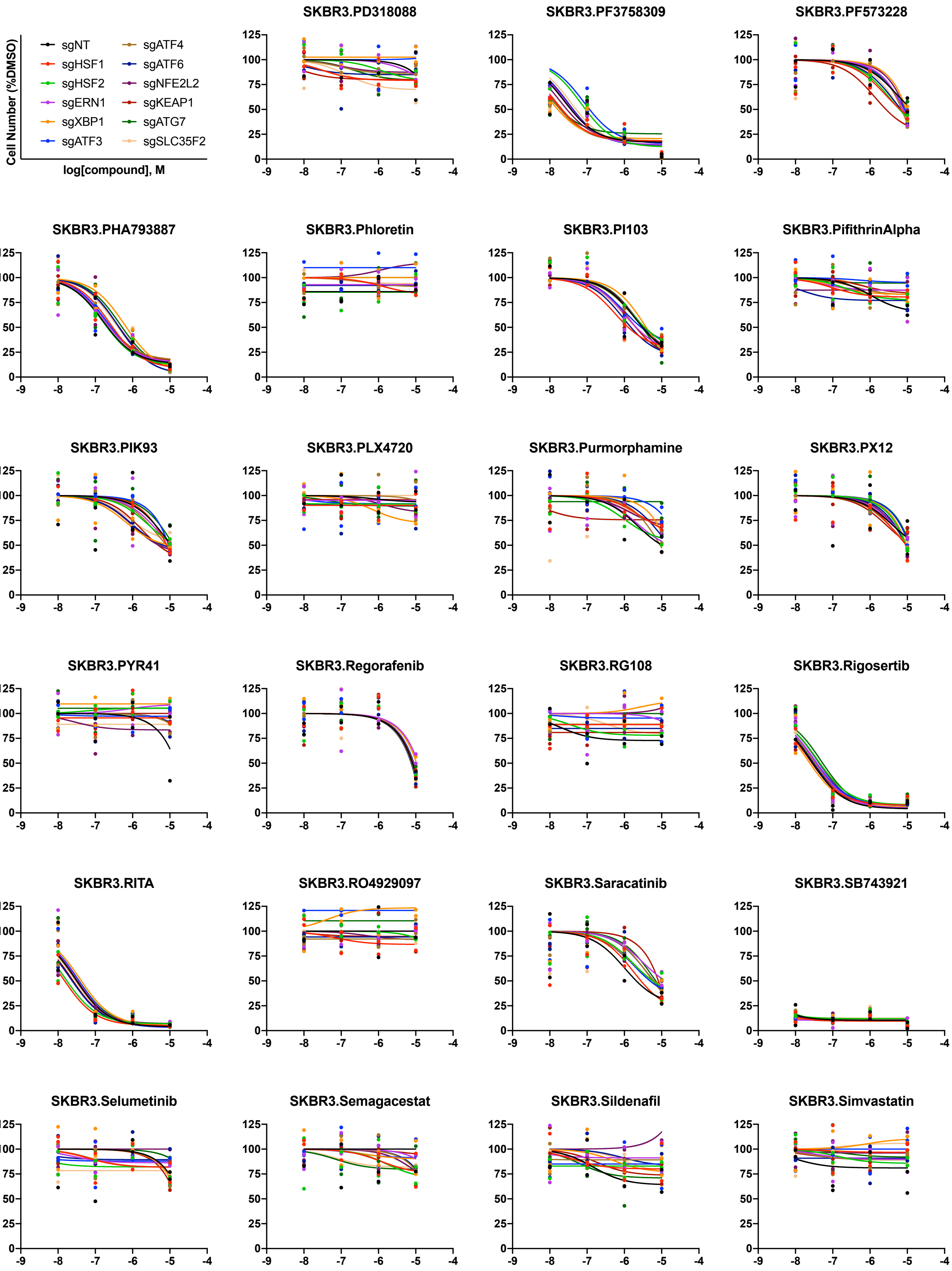

Supplementary Fig. 5 (continued). Dose-response curves generated using QMAP-Seq. Each plot represents a unique cell line-compound combination. Each data point represents one of two biologically independent replicates for the indicated sgRNA.

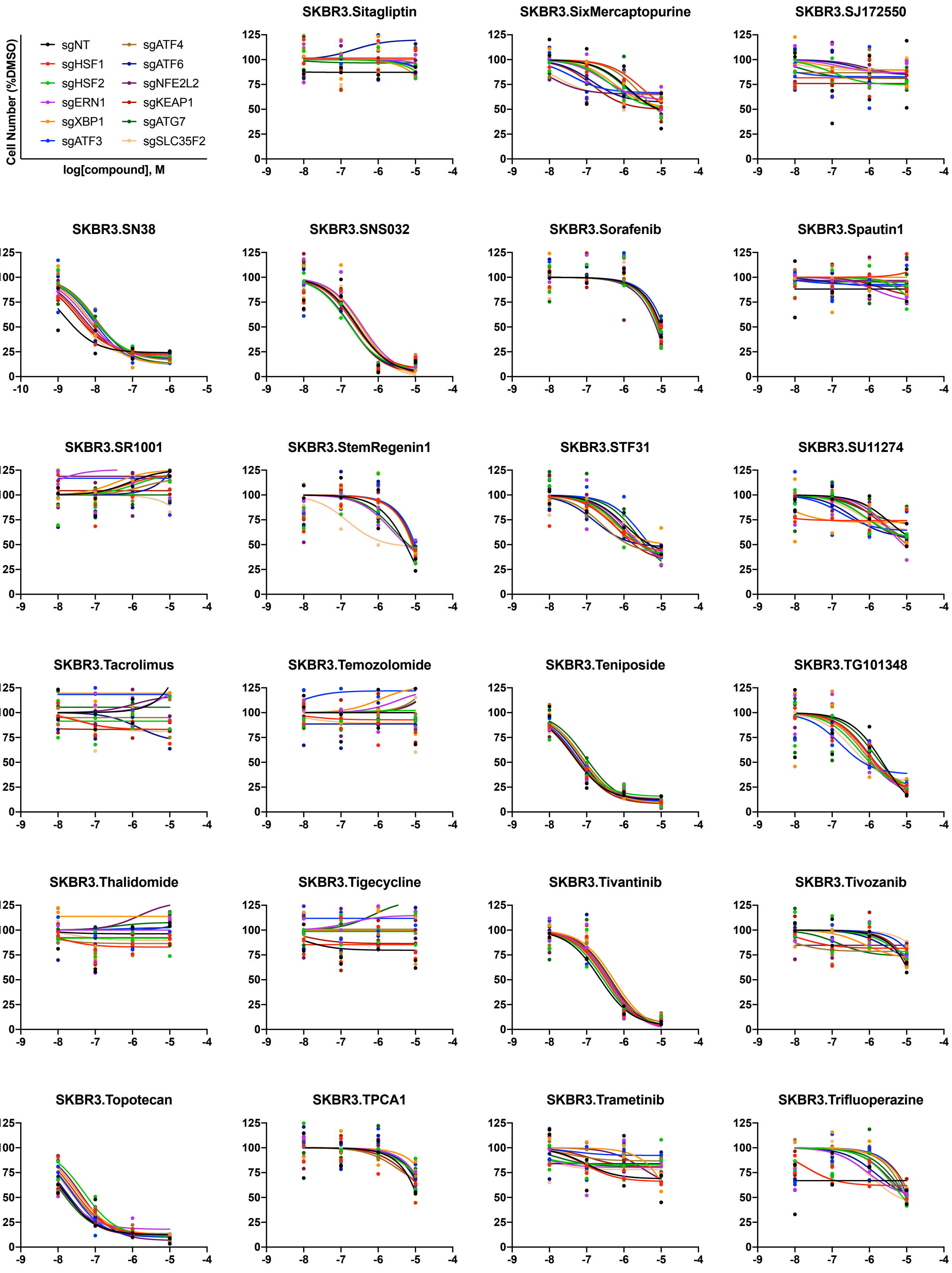

Supplementary Fig. 5 (continued). Dose-response curves generated using QMAP-Seq. Each plot represents a unique cell line-compound combination. Each data point represents one of two biologically independent replicates for the indicated sgRNA.

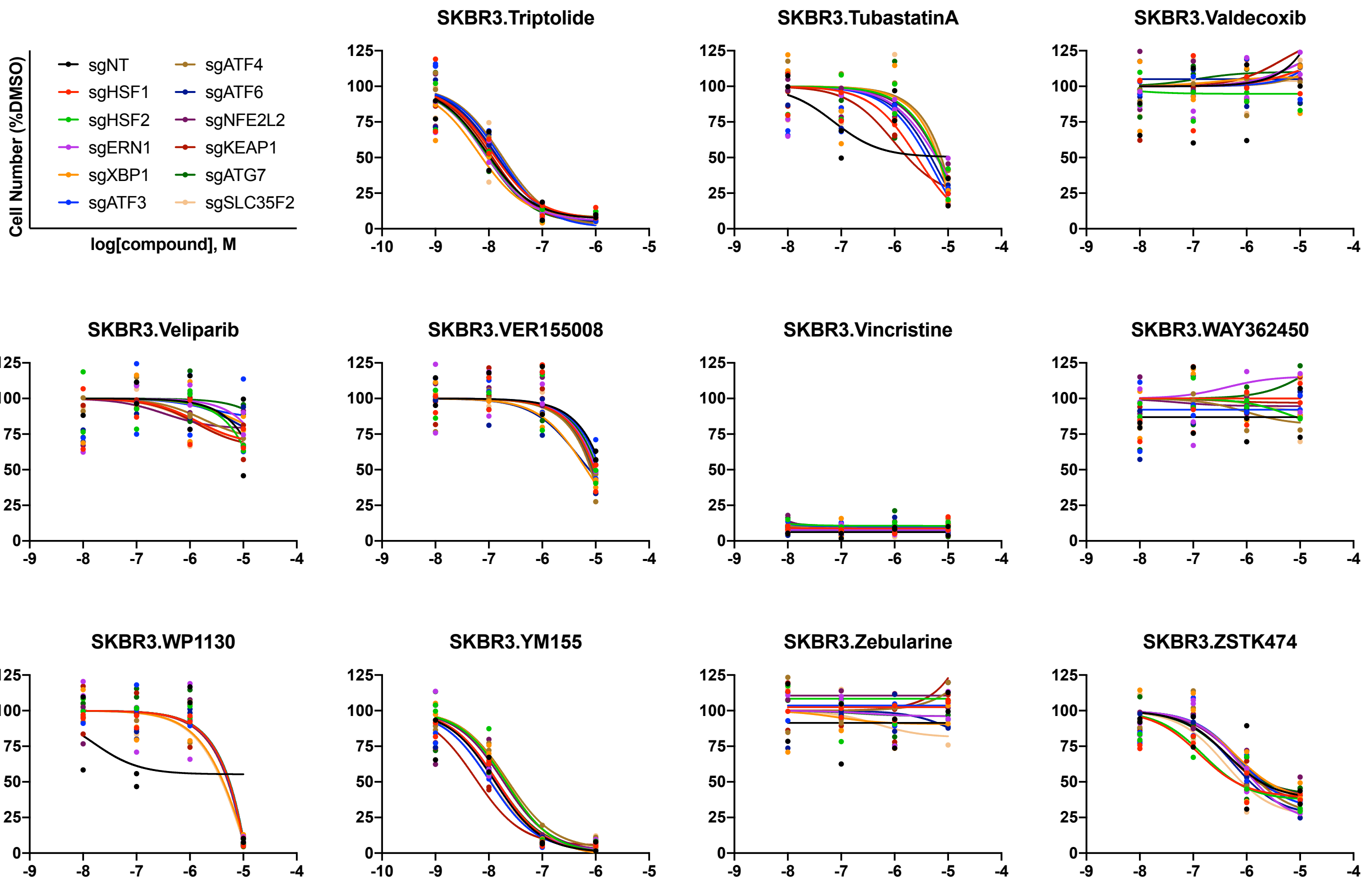

Supplementary Fig. 5 (continued). Dose-response curves generated using QMAP-Seq. Each plot represents a unique cell line-compound combination. Each data point represents one of two biologically independent replicates for the indicated sgRNA.

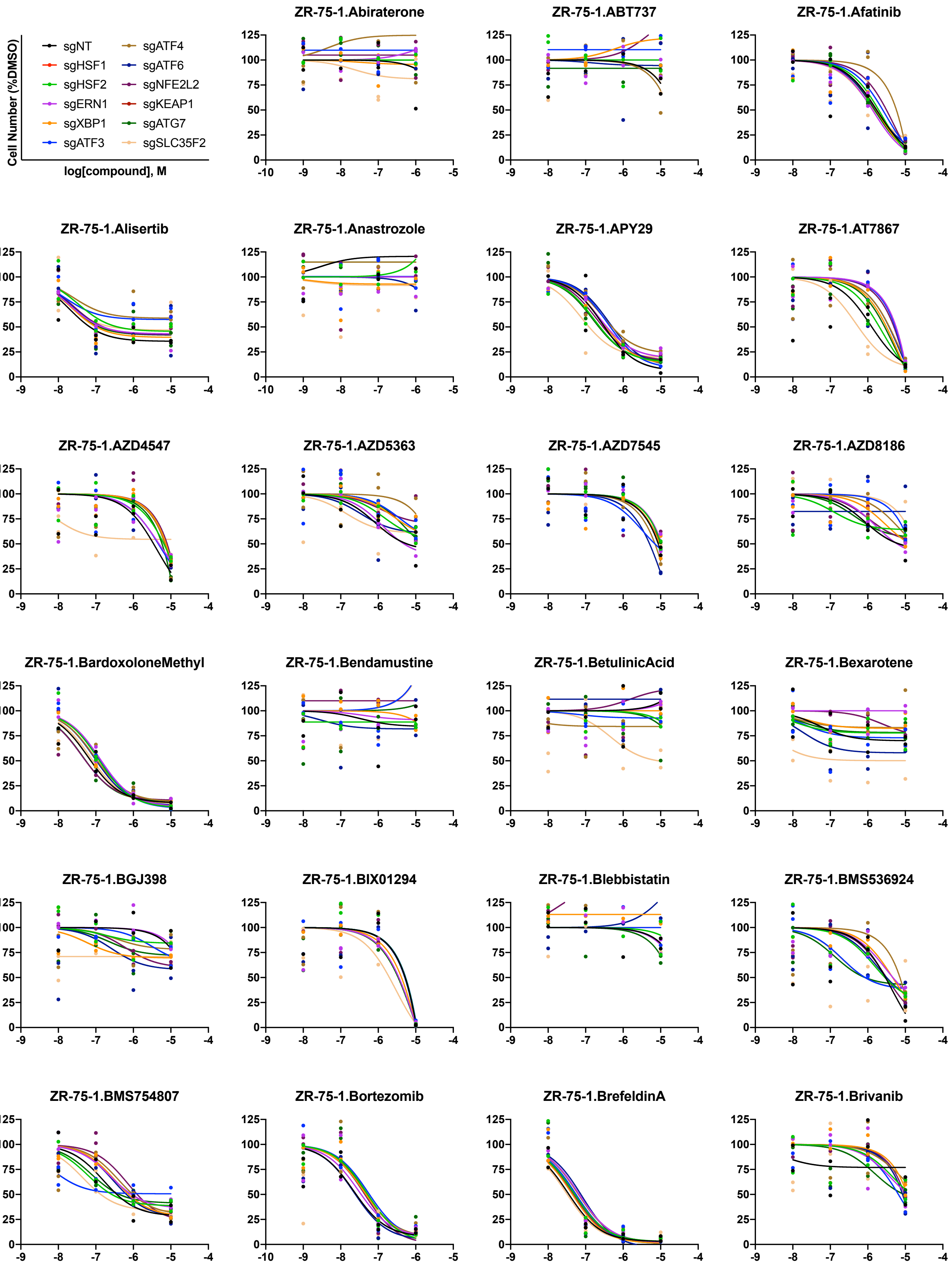

Supplementary Fig. 5 (continued). Dose-response curves generated using QMAP-Seq. Each plot represents a unique cell line-compound combination. Each data point represents one of two biologically independent replicates for the indicated sgRNA.

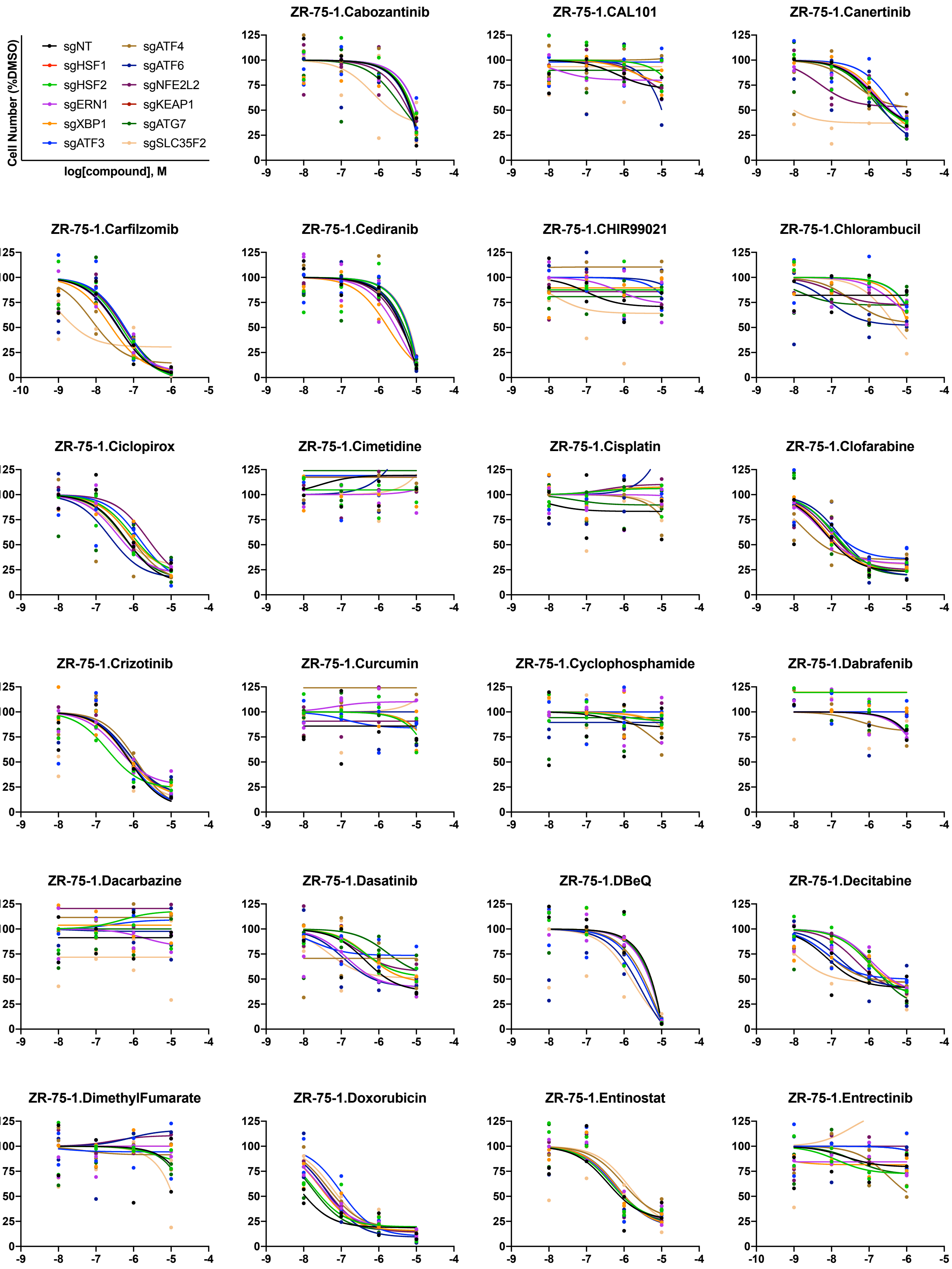

Supplementary Fig. 5 (continued). Dose-response curves generated using QMAP-Seq. Each plot represents a unique cell line-compound combination. Each data point represents one of two biologically independent replicates for the indicated sgRNA.

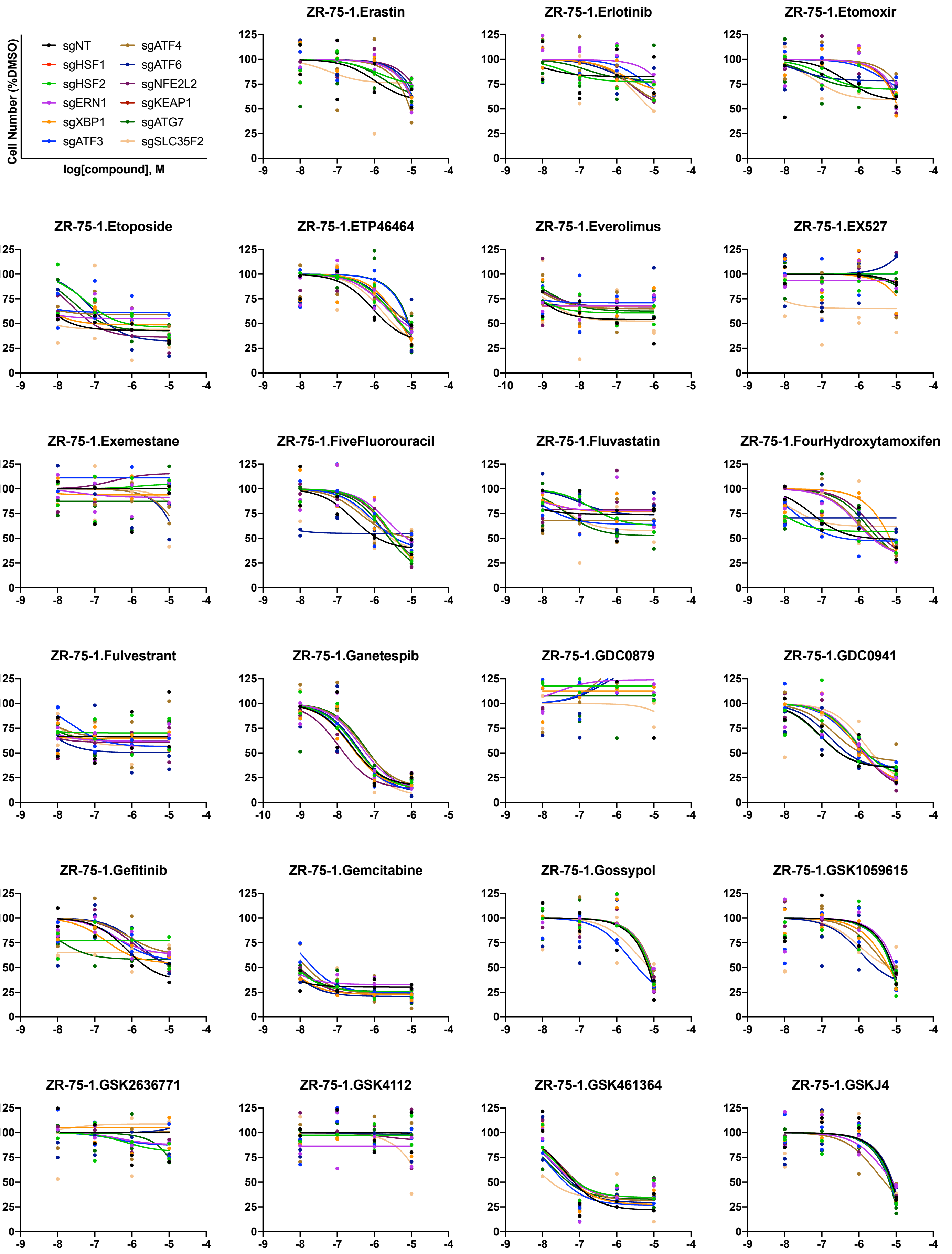

Supplementary Fig. 5 (continued). Dose-response curves generated using QMAP-Seq. Each plot represents a unique cell line-compound combination. Each data point represents one of two biologically independent replicates for the indicated sgRNA.

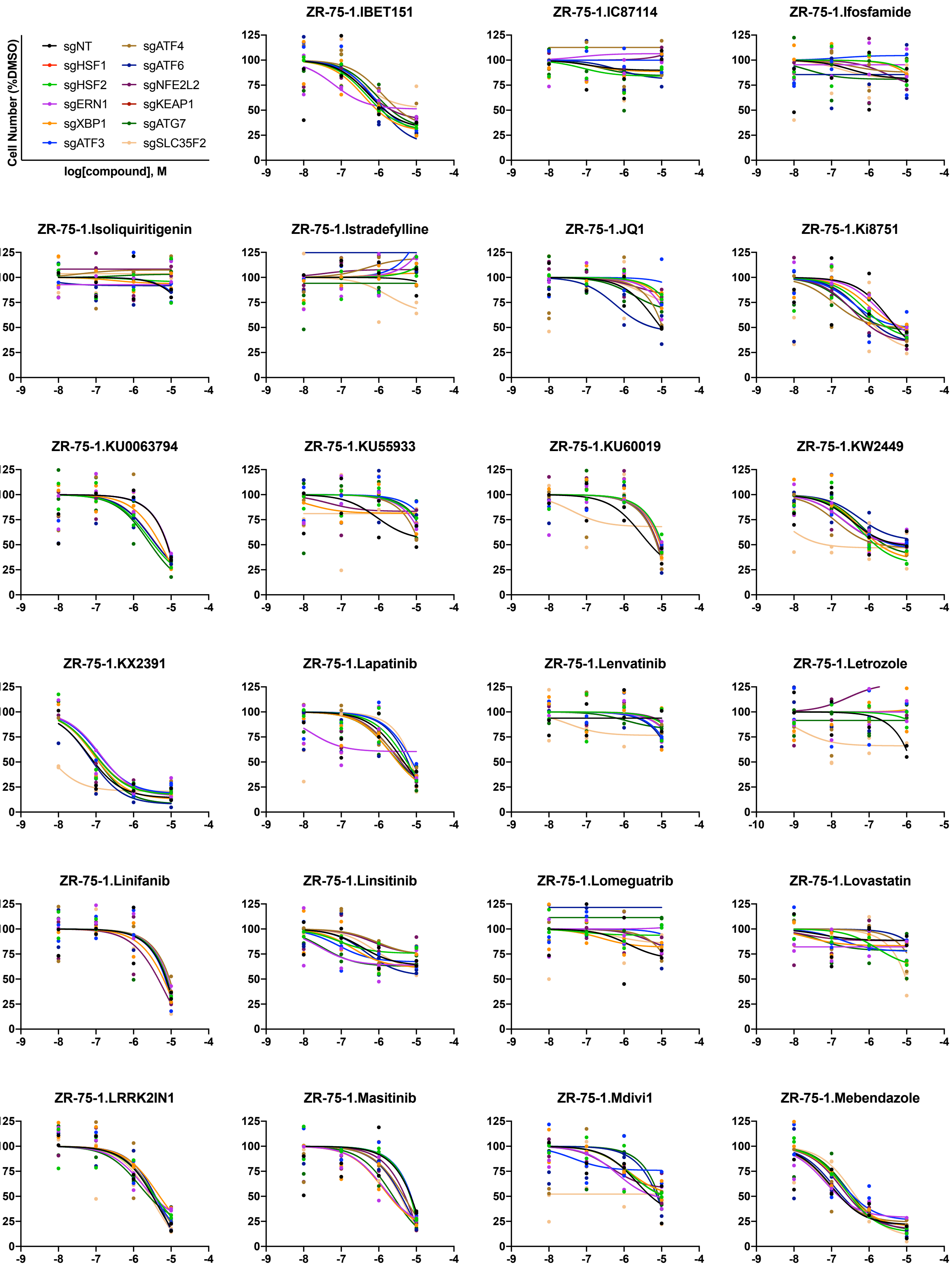

Supplementary Fig. 5 (continued). Dose-response curves generated using QMAP-Seq. Each plot represents a unique cell line-compound combination. Each data point represents one of two biologically independent replicates for the indicated sgRNA.

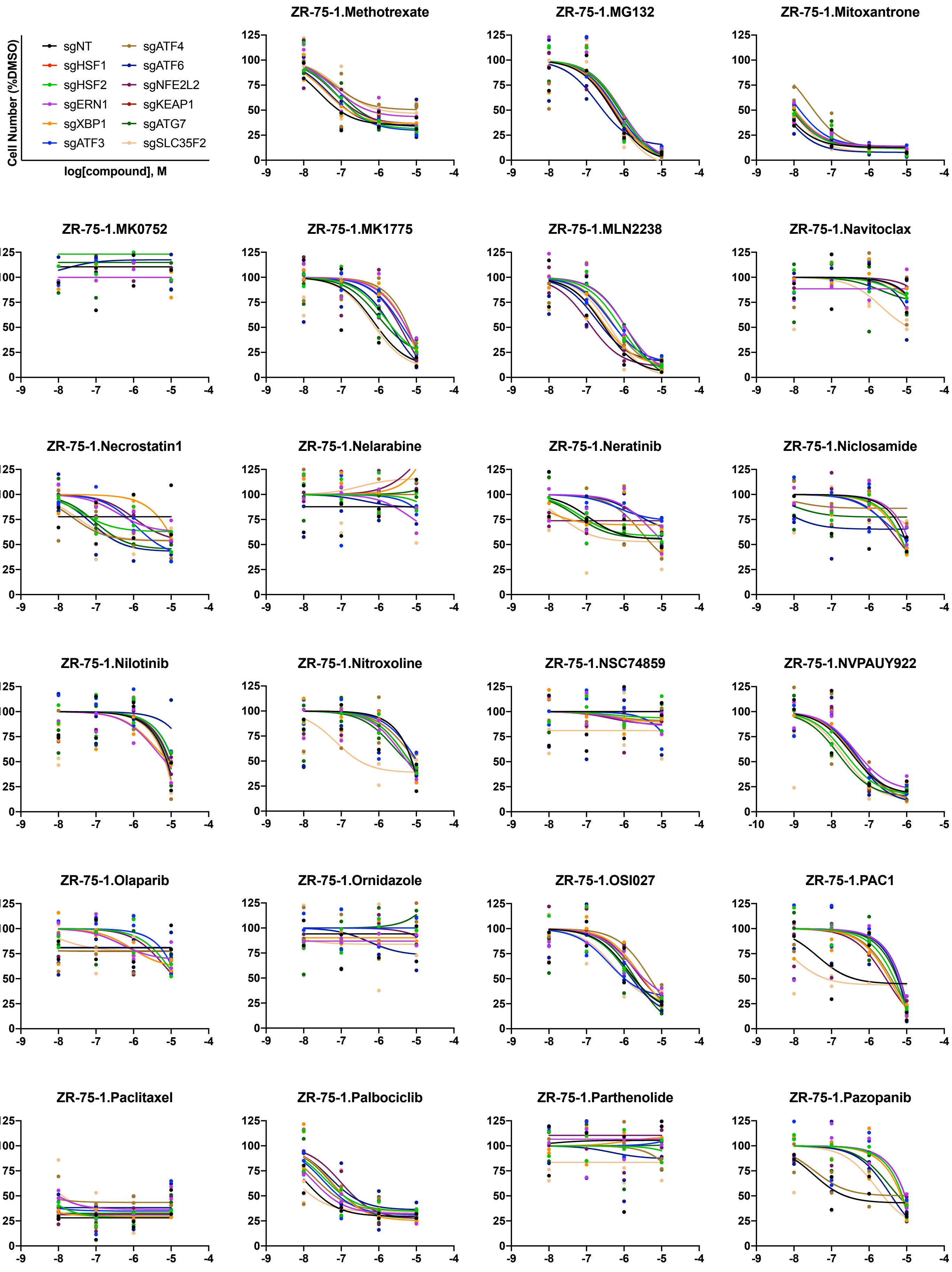

Supplementary Fig. 5 (continued). Dose-response curves generated using QMAP-Seq. Each plot represents a unique cell line-compound combination. Each data point represents one of two biologically independent replicates for the indicated sgRNA.

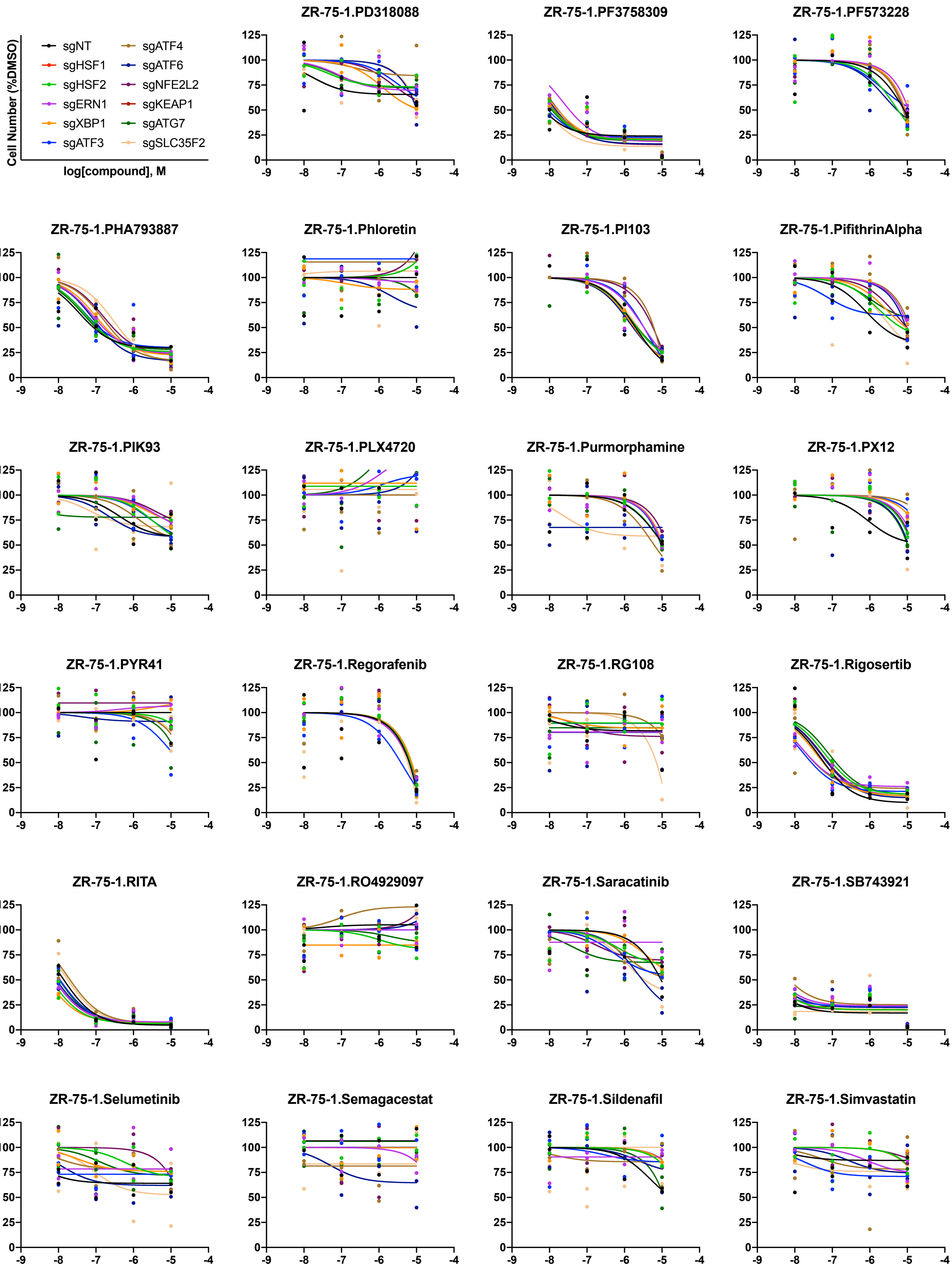

Supplementary Fig. 5 (continued). Dose-response curves generated using QMAP-Seq. Each plot represents a unique cell line-compound combination. Each data point represents one of two biologically independent replicates for the indicated sgRNA.

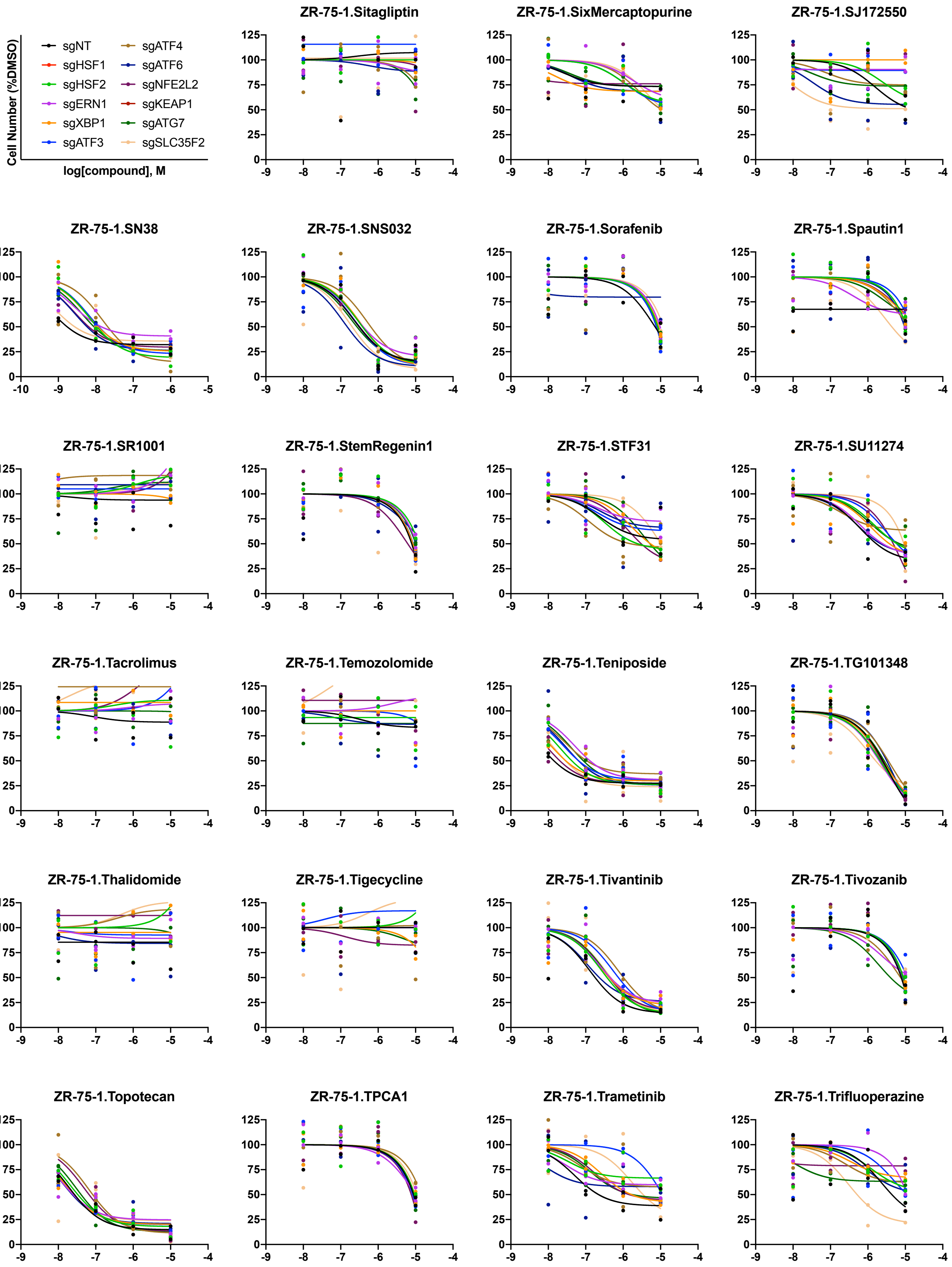

Supplementary Fig. 5 (continued). Dose-response curves generated using QMAP-Seq. Each plot represents a unique cell line-compound combination. Each data point represents one of two biologically independent replicates for the indicated sgRNA.

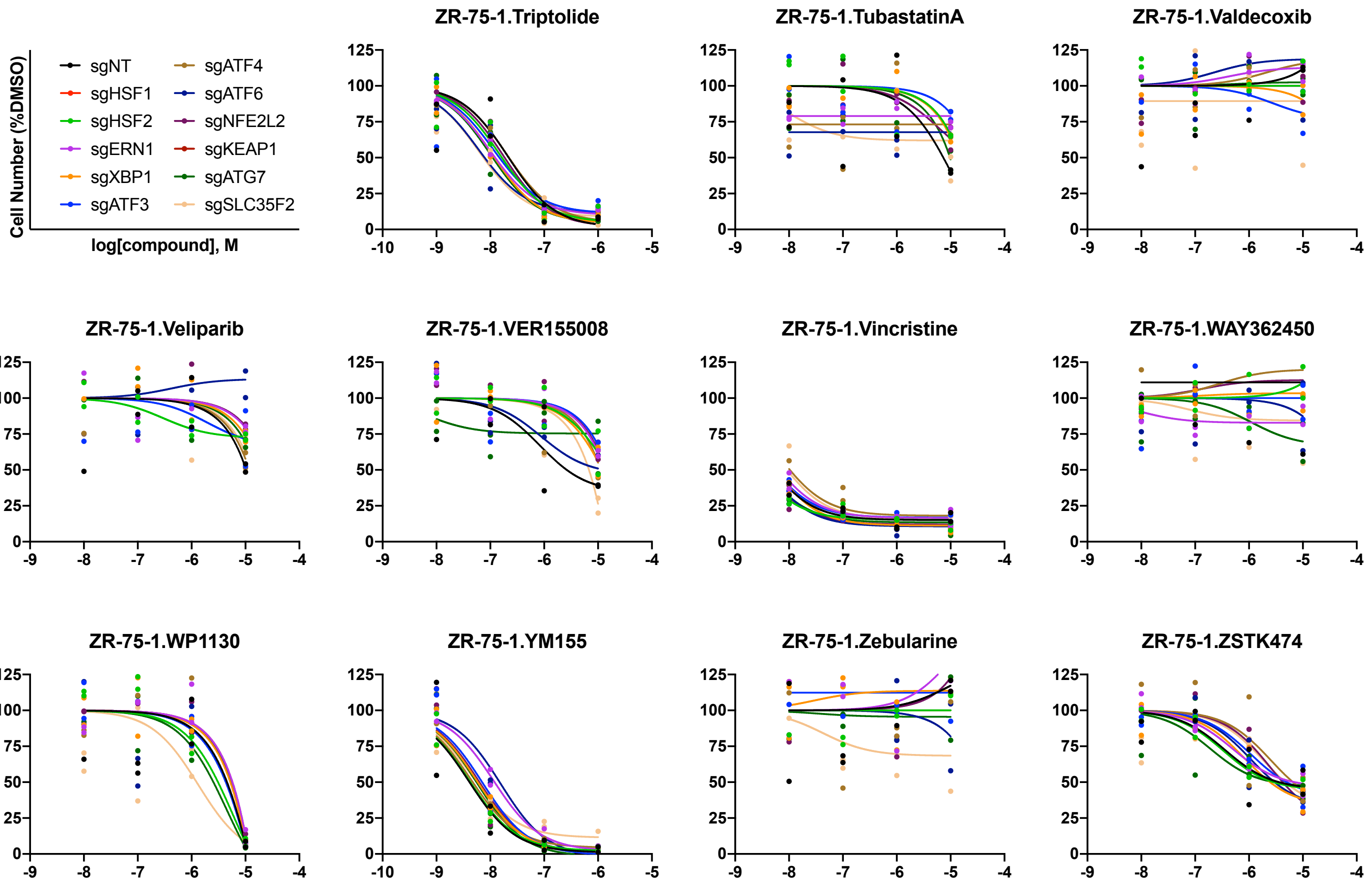

Supplementary Fig. 5 (continued). Dose-response curves generated using QMAP-Seq. Each plot represents a unique cell line-compound combination. Each data point represents one of two biologically independent replicates for the indicated sgRNA.
