# Supplementary material for: Quantitative and multiplexed chemical-genetic phenotyping in mammalian cells with QMAP-Seq
Source: Nat Commun. 2020 Nov 12;11:5722. doi: 10.1038/s41467-020-19553-8 (PMC7661543; doi:10.1038/s41467-020-19553-8)
Supplement: Supplementary file 2 — Description of Additional Supplementary Files [file 41467_2020_19553_MOESM2_ESM.pdf]

Title: Supplementary Data 1.
Descriptions: P5 5001-5016 staggered PCR primer sequences.

Title: Supplementary Data 2.
Descriptions: P7 7001-7096 PCR primer sequences.

Title: Supplementary Data 3.
Descriptions: i5 compound index sequences.

Title: Supplementary Data 4.
Descriptions: i7 compound index sequences.

Title: Supplementary Data 5.
Descriptions: Cell line barcode read sequences.

Title: Supplementary Data 6.
Descriptions: sgRNA barcode read sequences.

Title: Supplementary Data 7.
Descriptions: Compounds, compound targets, compound pathways, and dose ranges for QMAP-Seq.

Title: Supplementary Data 8.
Descriptions: Relative cell number values for the 83,520 cell line-sgRNA-compound-dose-replicate combinations.

Title: Supplementary Data 9.
Descriptions: AUC values for the 20,880 cell line-sgRNA-compound-replicate combinations.

Title: Supplementary Data 10.
Descriptions: AUC difference and significance values after data filtering. Statistical significance was determined using an unpaired, two-tailed t test ($n = 2$ biologically independent replicates). No adjustment was made for multiple comparisons.

Title: Supplementary Data 11.
Descriptions: Complete list of primers used for cloning and sequencing library amplification.
