# Supplementary material for: Quantitative and multiplexed chemical-genetic phenotyping in mammalian cells with QMAP-Seq
Source: Nat Commun. 2020 Nov 12;11:5722. doi: 10.1038/s41467-020-19553-8 (PMC7661543; doi:10.1038/s41467-020-19553-8)
Supplement: Supplementary file 15 — Reporting Summary [file 41467_2020_19553_MOESM15_ESM.pdf]

nature research

Corresponding author(s): Marc Mendillo

Last updated by author(s): Oct 11, 2020

Reporting Summary

Nature Research wishes to improve the reproducibility of the work that we publish. This form provides structure for consistency and transparency in reporting. For further information on Nature Research policies, see our Editorial Policies and the Editorial Policy Checklist.

Statistics

For all statistical analyses, confirm that the following items are present in the figure legend, table legend, main text, or Methods section.

n/a	Confirmed	
☐	☒	The exact sample size (*n*) for each experimental group/condition, given as a discrete number and unit of measurement
☐	☒	A statement on whether measurements were taken from distinct samples or whether the same sample was measured repeatedly
☐	☒	The statistical test(s) used AND whether they are one- or two-sided<br>*Only common tests should be described solely by name; describe more complex techniques in the Methods section.*
☒	☐	A description of all covariates tested
☐	☒	A description of any assumptions or corrections, such as tests of normality and adjustment for multiple comparisons
☐	☒	A full description of the statistical parameters including central tendency (e.g. means) or other basic estimates (e.g. regression coefficient) AND variation (e.g. standard deviation) or associated estimates of uncertainty (e.g. confidence intervals)
☐	☒	For null hypothesis testing, the test statistic (e.g. *F*, *t*, *r*) with confidence intervals, effect sizes, degrees of freedom and *P* value noted<br>*Give P values as exact values whenever suitable.*
☒	☐	For Bayesian analysis, information on the choice of priors and Markov chain Monte Carlo settings
☒	☐	For hierarchical and complex designs, identification of the appropriate level for tests and full reporting of outcomes
☐	☒	Estimates of effect sizes (e.g. Cohen's *d*, Pearson's *r*), indicating how they were calculated

Our web collection on statistics for biologists contains articles on many of the points above.

Software and code

Policy information about availability of computer code

Data collection	No software was used.
Data analysis	QMAP-Seq data was analyzed using custom code. Custom code is available at GitHub (https://github.com/mendillolab/QMAP-Seq) and Code Ocean (https://codeocean.com/capsule/3022355/tree/v1). Western blots were analyzed using Image Lab 5.2.1 (Bio-Rad). IncuCyte analysis was performed using IncuCyte ZOOM Live-Cell Analysis System GUI version 2015A (Essen BioScience). NGS samples were demultiplexed by running bcl2fastq2 Conversion Software v2.20 (Illumina). Heat maps were generated using Cluster 3.0 and Java TreeView 1.1.6r4. Statistical analysis was performed using GraphPad Prism 8.

For manuscripts utilizing custom algorithms or software that are central to the research but not yet described in published literature, software must be made available to editors and reviewers. We strongly encourage code deposition in a community repository (e.g. GitHub). See the Nature Research guidelines for submitting code & software for further information.

Data

Policy information about availability of data

All manuscripts must include a data availability statement. This statement should provide the following information, where applicable:

- Accession codes, unique identifiers, or web links for publicly available datasets
- A list of figures that have associated raw data
- A description of any restrictions on data availability

The data discussed in this publication, including raw fastq files, read counts, and relative cell numbers, have been deposited in NCBI's Gene Expression Omnibus and are accessible through GEO Series accession number GSE155855 (https://www.ncbi.nlm.nih.gov/geo/query/acc.cgi?acc=GSE155855). Source data for Fig. 1e, Fig. 2e, Supplementary Fig. 1b, and Supplementary Fig. 1d are provided with this paper.

Field-specific reporting

Please select the one below that is the best fit for your research. If you are not sure, read the appropriate sections before making your selection.

- [x] Life sciences
- [ ] Behavioural & social sciences
- [ ] Ecological, evolutionary & environmental sciences

For a reference copy of the document with all sections, see nature.com/documents/nr-reporting-summary-flat.pdf

Life sciences study design

All studies must disclose on these points even when the disclosure is negative.

Sample size	We selected a cell line pool size of 60 cell types (5 cell lines x 12 genetic perturbations) to enable adequate representation of each cell line-sgRNA combination in the pool (5,000 seeded cells / 60 cell types = 83 cells/cell type), while still enabling a large number of cell types to be profiled simultaneously in one well. We selected a sample size of two biologically independent replicates per compound-dose combination to enable assessment of reproducibility of our method (as shown in Fig. 1d), while still enabling a large number of compounds and doses to be tested in a single QMAP-Seq experiment (as each compound-dose-replicate condition required a unique sample barcode).
Data exclusions	For QMAP-Seq with one cell line, the data from Plate 8 was excluded from analysis due to a technical issue with the addition of cell spike-in standards for that plate. For QMAP-Seq with multiple cell lines, data was excluded if it did not pass the following quality-control measures. First, samples with low read counts were excluded due to insufficient data quantity. Second, cell line-sgRNA pairs with high variation (standard deviation of log2(cell number) > 0.7) were excluded due to low data quality. Third, compounds with an AUC > 550 for any cell line-sgRNA pair were excluded due to concerns over data quality specific to well B7. Fourth, noncytotoxic cell line-compound pairs (relative cell number of sgNT with highest dose > 90%) were excluded due to the inability to capture the appropriate dose range. Fifth, cell line-compound pairs without a dose-dependent reduction in cell viability (difference in relative cell number of sgNT between lowest and highest dose < 25%) were excluded due to the lack of a dose-dependent response. These exclusion criteria were not pre-established.
Replication	Assessment of FLAG-Cas9 induction by Western blotting was performed twice in independent experiments. Confirmation of proteostasis factor knockout by Western blotting was performed once. IncuCyte live-cell imaging was performed using two biologically independent replicates. Each QMAP-Seq experiment was performed using two biologically independent replicates, which were found to be correlated (Fig. 1d). The results from the 220 common compound-sgRNA combinations between the two independent QMAP-Seq experiments were also reproducible (Fig. 3b). Resazurin-based validation was performed using three biologically independent replicates.
Randomization	Compound treatment responses were measured for all cell lines in a cell line pool, so there was no need to assign samples to control or treatment groups.
Blinding	Blinding was not relevant to this study because all cell lines were measured in both control and treatment groups. Furthermore, all measurements were quantitative measurements of cell number that were read out by a next-generation sequencing instrument rather than subjective measurements.

Reporting for specific materials, systems and methods

We require information from authors about some types of materials, experimental systems and methods used in many studies. Here, indicate whether each material, system or method listed is relevant to your study. If you are not sure if a list item applies to your research, read the appropriate section before selecting a response.

Materials & experimental systems

n/a	Involved in the study
[ ]	[x] Antibodies
[ ]	[x] Eukaryotic cell lines
[x]	[ ] Palaeontology and archaeology
[x]	[ ] Animals and other organisms
[x]	[ ] Human research participants
[x]	[ ] Clinical data
[x]	[ ] Dual use research of concern

Methods

n/a	Involved in the study
[x]	[ ] ChIP-seq
[ ]	[x] Flow cytometry
[x]	[ ] MRI-based neuroimaging

Antibodies

Antibodies used	FLAG (Sigma-Aldrich, #F3165, Clone M2)<br>HSF1 (Santa Cruz Biotechnology, #sc-9144, Clone H-311)<br>HSF2 (Santa Cruz Biotechnology, #sc-13517, Clone 3E2)<br>IRE1 (Cell Signaling Technology, #3294, Clone 14C10)<br>XBP1 (Cell Signaling Technology, #12782, Clone D2C1F)<br>ATF3 (abcam, #ab207434, Clone EPR19488)<br>ATF4 (Cell Signaling Technology, #11815, Clone D4B8)

	ATF6 (Cell Signaling Technology, #65880, Clone D4Z8V)<br>ATG7 (Cell Signaling Technology, #8558, Clone D12B11)<br>NRF2 (Cell Signaling Technology, #12721, Clone D1Z9C)<br>KEAP1 (Cell Signaling Technology, #4617, Clone H436)<br>Alpha Tubulin (abcam, #ab80779, Clone DM1A)<br>Beta Actin (Thermo Fisher Scientific, #MA5-15739, Clone BA3R)<br>Anti-Mouse IgG-Peroxidase secondary antibody (Sigma-Aldrich, #A9044, polyclonal)
Validation	Primary antibodies were validated for Western blotting with human species reactivity as noted on the manufacturers' websites:<br>FLAG (Sigma-Aldrich, #F3165, Clone M2): Antibody was used in at least 4,956 publications, including for human species Western blotting.<br>HSF1 (Santa Cruz Biotechnology, #sc-9144, Clone H-311): Antibody was used in at least 14 publications, including for human species Western blotting.<br>HSF2 (Santa Cruz Biotechnology, #sc-13517, Clone 3E2): Antibody was used in at least 3 publications, including for human species Western blotting.<br>IRE1 (Cell Signaling Technology, #3294, Clone 14C10): Antibody was used specifically for human species Western blotting in at least 33 publications.<br>XBP1 (Cell Signaling Technology, #12782, Clone D2C1F): Antibody was used specifically for human species Western blotting in at least 10 publications.<br>ATF3 (abcam, #ab207434, Clone EPR19488): Antibody was used in at least 10 publications, including for human species Western blotting.<br>ATF4 (Cell Signaling Technology, #11815, Clone D4B8): Antibody was used specifically for human species Western blotting in at least 31 publications.<br>ATF6 (Cell Signaling Technology, #65880, Clone D4Z8V): Antibody was used specifically for human species Western blotting in at least 6 publications.<br>ATG7 (Cell Signaling Technology, #8558, Clone D12B11): Antibody was used specifically for human species Western blotting in at least 23 publications.<br>NRF2 (Cell Signaling Technology, #12721, Clone D1Z9C): Antibody was used specifically for human species Western blotting in at least 12 publications.<br>KEAP1 (Cell Signaling Technology, #4617, Clone H436): Antibody was used specifically for human species Western blotting in at least 1 publication.<br>Alpha Tubulin (abcam, #ab80779, Clone DM1A): Antibody was used in at least 39 publications, including for human species Western blotting.<br>Beta Actin (Thermo Fisher Scientific, #MA5-15739, Clone BA3R): Antibody was used in at least 196 publications, including for human species Western blotting.

Eukaryotic cell lines

Policy information about cell lines

Cell line source(s)	293T, ZR-75-1, SKBR3, HCC-38, MDA-MB-231, and BT-20 cells were obtained from ATCC.
Authentication	All cell lines were authenticated at the University of Arizona Genetics Core using autosomal STR profiling.
Mycoplasma contamination	All cell lines tested negative for mycoplasma contamination.
Commonly misidentified lines<br>(See ICLAC register)	BT-20 was initially thought to be a commonly misidentified cell line, but an authentic stock has since been found at ATCC. We obtained our cells from ATCC and further authenticated these cells using autosomal STR profiling. We were not aware that this cell line was previously considered a commonly misidentified cell line when we included it in our breast cancer cell line panel.

Flow Cytometry

Plots

Confirm that:

- [x] The axis labels state the marker and fluorochrome used (e.g. CD4-FITC).
- [x] The axis scales are clearly visible. Include numbers along axes only for bottom left plot of group (a 'group' is an analysis of identical markers).
- [x] All plots are contour plots with outliers or pseudocolor plots.
- [x] A numerical value for number of cells or percentage (with statistics) is provided.

Methodology

Sample preparation	We prepared five cell line pools, each containing 20% of one cell line expressing ZsGreen and 20% of each of the other four cell lines expressing dTomato. At each time point, we pelleted 1,000,000 cells/pool and resuspended in 400 uL media prior to flow cytometry analysis.
Instrument	LSRFortessa Cell Analyzer (BD Biosciences)

Software	FACSDiva 6.1.3 (BD Biosciences)
Cell population abundance	Our analytical flow cytometry did not involve cell sorting.
Gating strategy	First, cells were gated using FSC-A vs. SSC-A to eliminate debris and dead cells. Second, cells were gated using SSC-W vs. FSC-H followed by FSC-W vs. SSC-H to eliminate doublets. Finally, cells were gated using Alexa Fluor 488 vs. PE-YG to quantify the percentage of GFP+ and RFP+ cells, respectively. The raw data (plots) and gating strategy related to Fig. 2e are provided as a Source Data file.

☒ Tick this box to confirm that a figure exemplifying the gating strategy is provided in the Supplementary Information.
